# Improving the Measurement of Semantic Similarity between Gene Ontology Terms and Gene Products: Insights from an Edge- and IC-Based Hybrid Method

**DOI:** 10.1371/journal.pone.0066745

**Published:** 2013-05-31

**Authors:** Xiaomei Wu, Erli Pang, Kui Lin, Zhen-Ming Pei

**Affiliations:** 1 College of Life and Environmental Sciences, Hangzhou Normal University, Hangzhou, People's Republic of China; 2 Laboratory for Biodiversity Science and Ecological Engineering, College of Life Sciences, Beijing Normal University, Beijing, People's Republic of China; 3 Department of Biology, Duke University, Durham, North Carolina, United States of America; Semmelweis University, Hungary

## Abstract

**Background:**

Explicit comparisons based on the semantic similarity of Gene Ontology terms provide a quantitative way to measure the functional similarity between gene products and are widely applied in large-scale genomic research via integration with other models. Previously, we presented an edge-based method, Relative Specificity Similarity (RSS), which takes the global position of relevant terms into account. However, edge-based semantic similarity metrics are sensitive to the intrinsic structure of GO and simply consider terms at the same level in the ontology to be equally specific nodes, revealing the weaknesses that could be complemented using information content (IC).

**Results and Conclusions:**

Here, we used the IC-based nodes to improve RSS and proposed a new method, Hybrid Relative Specificity Similarity (HRSS). HRSS outperformed other methods in distinguishing true protein-protein interactions from false. HRSS values were divided into four different levels of confidence for protein interactions. In addition, HRSS was statistically the best at obtaining the highest average functional similarity among human-mouse orthologs. Both HRSS and the groupwise measure, simGIC, are superior in correlation with sequence and Pfam similarities. Because different measures are best suited for different circumstances, we compared two pairwise strategies, the maximum and the best-match average, in the evaluation. The former was more effective at inferring physical protein-protein interactions, and the latter at estimating the functional conservation of orthologs and analyzing the CESSM datasets. In conclusion, HRSS can be applied to different biological problems by quantifying the functional similarity between gene products. The algorithm HRSS was implemented in the C programming language, which is freely available from http://cmb.bnu.edu.cn/hrss.

## Introduction

With the advent of high-throughput technologies such as DNA and RNA sequencing and microarray, automatic genome annotation of large sets of genes has been increasingly used. An accessible and systematic scheme is required to handle the large amount of annotations to genes and their products, and to make comparisons of these gene products computationally interpretable on a standard platform. The Gene Ontology (GO) [1] system is one such scheme that is widely becoming the *de facto* standard for facilitating information searches across databases and for aiding the annotation of molecular features in different model organisms. GO has been successfully used in protein classification [2], [3], prediction and validation of protein-protein interactions [4], [5], gene expression studies [6] and homology analysis [7].

GO consists of three structured ontologies which allow the description of molecular function (MF), biological process (BP), and cellular component (CC). Each ontology is structured as a directed acyclic graph (DAG), which differs from hierarchies in that a ‘child’, a more specialized term, can have many ‘parents’ or ‘ancestors’, a less specialized or more general term. Child terms are instances or components of parent terms. Therefore, GO follows a true path rule where annotation to a given term implies annotation to all of its ancestors by inheritance. The valuable functional knowledge encoded in GO should be useful for developing new predictive systems to compare gene products at the functional level, which may be integrated with other models in large-scale genomic research.

The functional relationships between gene products annotated with GO terms are quantified either implicitly using methods for the shared GO terms of gene products [5], [8]–[10] or explicitly using semantic similarity measures. The former method is restricted to protein pairs with the same annotations; the semantic similarity measure is more commonly used and returns a numerical value quantifying the relationship between two GO terms or two sets of terms annotating two gene products. Given two terms, GO-based semantic similarity measures may be classified into edge- and node-based groups; given two gene products, the measures may be classified into pairwise and groupwise approaches [11].

Edge-based methods determine semantic similarity based primarily on counting the number of edges (*distance*) along the paths linking the GO terms being considered. The specificity of the most recent common ancestor (MRCA) of two GO terms, measured by the distance between the ancestor and the root term in the GO DAG, was used as the only parameter to quantify the semantic similarity between the term pair [12], [13]. But the MRCA only focuses on the hierarchy above the given terms and misses the hierarchy information below the given terms. To take the global structure of GO DAG into account, we designed a Relative Specificity Similarity (RSS) method that was explicitly based upon Wu's measure [12], and also includes the distance of the given term pair to its closest leaf terms (the generality of the two terms) and the distance to their MRCA [4]. However, edge-based semantic similarity metrics also have weaknesses. They use the GO DAG topology to compute the similarity, and as a result they are sensitive to the intrinsic structure of the GO DAG. Furthermore, they simply consider terms at the same level in the ontology to be equally specific nodes and edges at the same level to correspond to the same semantic distance between two terms, both of which are seldom true in biological ontologies [11].

Node-based approaches rely on comparing the properties of the terms involved and their ancestors or descendants. The property of a GO term can be estimated by a variety of means [14]–[16]. Information content (IC) is commonly used to estimate the property of a term *c*, as well as to measure how specific and informative the term is. Resnik measures (called ‘Resnik’ in this study) the similarity between two terms as simply the IC of their most informative common ancestor (MICA), indicating its specificity [17]. Three additional measures by Jiang and Conrath [18] (called ‘Jiang’), Lin [19] (called ‘Lin’) and Schlicker et al. [20] are different variants of Resnik. Couto et al. proposed the GraSM approach, which replaced the IC of the MICA with the average IC of all disjunctive common ancestors (DCAs) [21], and then exploited DiShIn to update GraSM [22]. Recently, Jain and Bader have developed the Topological Clustering Semantic Similarity (TCSS) method by considering the unequal depth of biological knowledge represented in different branches of the GO DAG [23]. Yang et al. improved the semantic similarity between two terms by considering not only their common ancestors but also their common descendants [24].

All of these methods quantify the relationship between two gene products by using pairwise strategies, such as the average, maximum and beset-match average, based on measuring every term pair. Other methods, such as simUI [25], simGIC [26] and the more recently designed SORA [27], calculate the semantic similarity between two gene products based on measuring two sets of annotated terms and are named as groupwise measures. Evaluations of similarity in sequence or gene expression are needed to assess how well a given measure represents the similarity between gene products. Both TCSS and Resnik with the maximum strategy are more suitable for predicting and validating protein-protein interactions and for correlating with gene expression [23], [28], [29]. SimGIC and Resnik (with the best-match average) have the highest correlation with sequence similarity [30], [31]. Besides, Resnik performs best in characterizing human regulatory pathways using the average strategy [32]. Therefore, no measure is clearly preferred over the others for biological problems; different measures are best suited in different contexts [11], [33].

IC-based metrics are less sensitive to the problem of variable semantic distances compared with edge-based approaches [17]. Moreover, Node-based methods highlight the idea that the terms on the same level of the GO DAG are not always equivalent because their importance or specificity in GO is measured by IC. Therefore, the edges at the same level do not always have equal semantic distances between two terms, a reasonable conclusion in biological ontology. In this study, we aimed to improve the RSS edge-based method that we designed previously, by employing the concept of information content. The new semantic similarity algorithm, called Hybrid Relative Specificity Similarity (HRSS), was a hybrid version between edge- and IC-based concepts. Four independent evaluation systems were then developed to analyze the performance of HRSS against the following methods: RSS; four commonly used nodes-based measures, Resnik, Jiang, Lin and TCSS with the maximum (MAX) and best-match average (BMA) strategies; and two groupwise measures, simUI and simGIC. The points of evaluation included (i) scoring protein-protein interactions in yeast and human using receiver operating characteristic (ROC) analysis, (ii) quantifying the functional similarity between human-mouse orthologs, and (iii) correlating the semantic similarities between UniProt proteins with the similarities of sequences, Pfam domains and Enzyme Commission classes (ECC) based on the platform of Collaborative Evaluation of Semantic Similarity Measures (CESSM) [34]. Furthermore, we carefully defined reasonable HRSS cutoff values for capturing high-quality positive and negative protein interactions, and then divided HRSS values into four groups with different confidence levels in BP and CC ontologies. The performance of two pairwise strategies, MAX and BMA, were also evaluated and compared in different evaluation systems.

## Materials and Methods

### Algorithm

We designed an edge-based semantic similarity method, RSS [4], which took both specificity (the hierarchy above the given terms) and generality (the hierarchy below the given terms) of the terms into account. Due to the drawback of edge-based measures, we used information content from node-based measures and combined it with the structure of RSS. In this section, we describe the RSS method first, and then present the new method.

#### The previously designed Relative Specificity Similarity method

**Figure 1:**
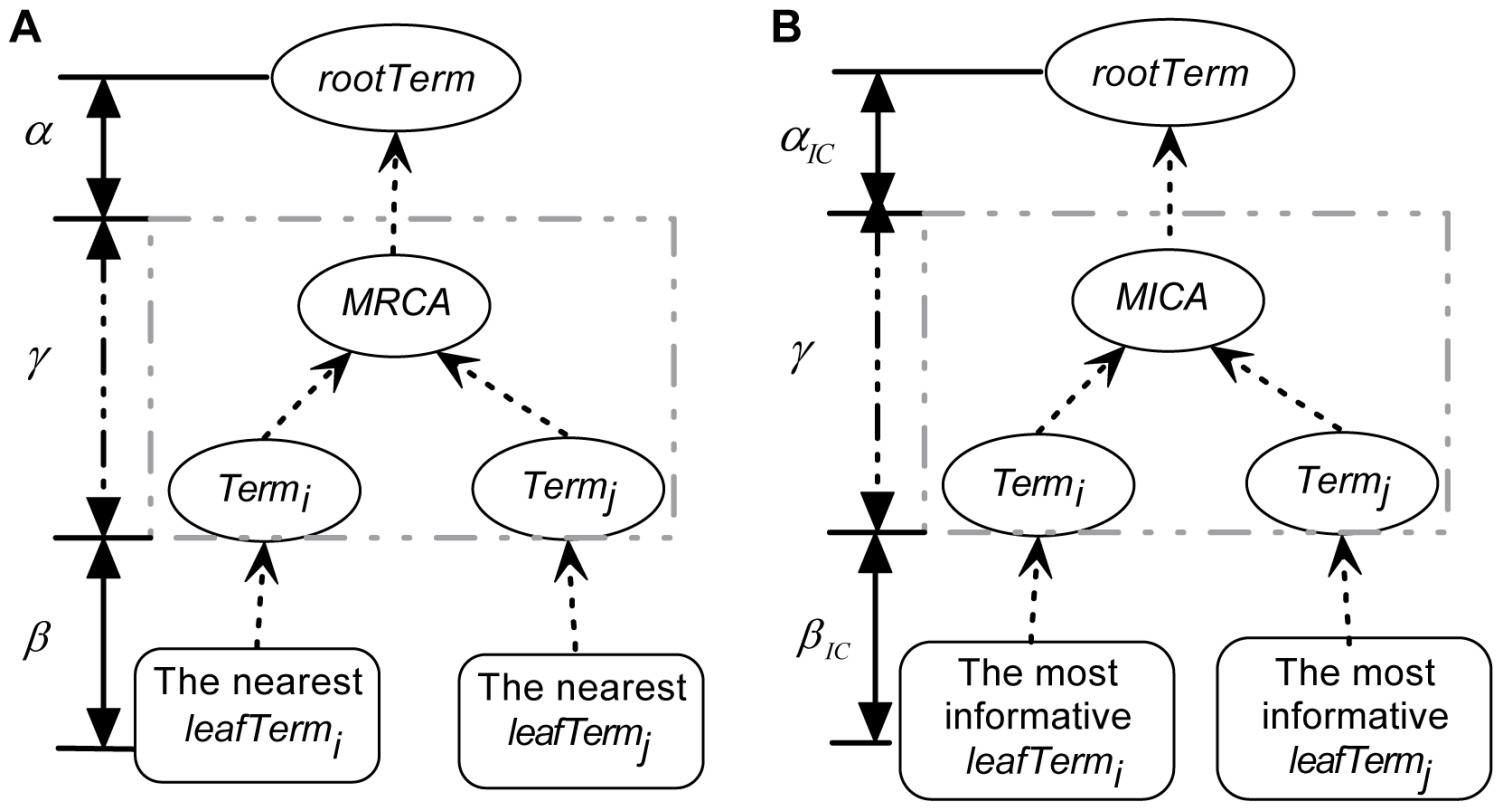
A schematic illustration of our algorithms. (**A**) Relative Specificity Similarity (RSS) is an edge-based method, but (**B**) Hybrid Relative Specificity Similarity (HRSS) integrates information content within the framework of RSS. Both of MRCA, the most recent common ancestor of *term_i_* and *term_j_*, and the MICA, the most informative common ancestor, represent the most specific term among all common ancestors of the term pair but are calculated in different ways. HRSS (RSS) consists of three components: *α_IC_* (*α*) represents the specificity of MICA (MRCA), *β_IC_* (*β*) indicates the generality of *term_i_* and *term_j_* in GO, and *γ* measures the local distance of *term_i_* and *term_j_* from their common ancestor.

For a given GO, let *term_i_* and *term_j_* be two terms. We defined *dist(term_i_, term_j_)* as the number of edges along the shortest path between these two terms, such that its value equals zero if *term_i_* and *term_j_* are the same term. The RSS of *term_i_* and *term_j_* consists of three different components (Figure 1A), denoted *α*, *β* and *γ*. Component *α* measures how specific the most recent common ancestor (MRCA) of the two terms is according to the structure of the GO. Component *β* measures how general *term_i_* and *term_j_* are in the GO. The generality of a term is defined as the minimum distance between the term and the leaf terms descending from it. Component *γ* measures the local distance between the two terms and their MRCA (Formula 1),
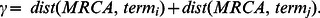
(1)


Then, the RSS between the two terms of a given GO, *term_i_* and *term_j_* can be quantified by combining *α*, *β* and *γ* together,
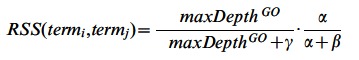
(2)where *maxDepth^GO^* is the maximum distance from the root term of the GO to the leaf terms. From the definition, the values of RSS are between 0 and 1. See Section S1 in Text S1 or the original study [4] for more details about the algorithm.

#### Hybrid Relative Specificity Similarity of two proteins annotated in a GO

IC is defined as the negative log likelihood of a term *c*,
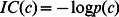
(3)where *p(c)* is the probability of occurrence of the term *c* in a specific corpus, such as the GO annotations of the yeast genome or the UniProt Knowledgebase, and is normally measured by the frequency of the number of genes annotated to *c* and all its descendants over the total number of genes in the corpus. The more often the term is used for annotation, the lower its semantic value.

We defined the IC-based distance between two terms *u* and *v*, where *u* is an ancestor of v, as the difference between their IC values,
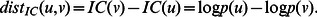
(4)


Then, the IC-based specificity of the most recent common ancestor of any two terms, *term_i_* and *term_j_* (i.e. component *α* in RSS method) is 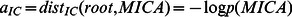
(5)where MICA is the most informative common ancestor of *term_i_* and *term_j_*. Resnik directly uses *α_IC_* to score the semantic similarity between the two terms [17].

The IC-based generality of a term is defined as the *dist_IC_* between the term and the most informative leaf nodes (MIL) descending from it. It is likely that not all of its leaf terms have proteins annotated in a particular corpus. Therefore, only the leaf terms with annotations were considered. A new component *β* is defined as the average of the generality values of *term_i_* and *term_j_*,
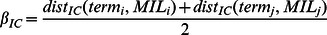
(6)where *MIL_i_* and *MIL_j_* are the most informative leaf nodes of *term_i_* and *term_j_*, respectively.

An improved RSS method adapting both node- and edge-based concepts, called Hybrid Relative Specificity Similarity (HRSS) (Figure 1B), is based on the framework of the RSS algorithm,
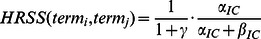
(7)where the definition of component *γ* is similar to that in the RSS method. By replacing the MRCA in Formula 1 with the MICA, we have 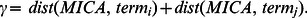
(8)Let *P* and *Q* be two gene products of interest, and *TP* and *TQ* the sets of all the GO terms assigned to proteins *P* and *Q*, respectively. The relationship strength between *P* and *Q* is defined through two pairwise rules, maximum (MAX) and best-match average (BMA). The MAX strategy takes the maximum semantic similarity value of all term pairs between TP and TQ as gene functional similarity (Formula 9), whereas the BMA strategy finds all the best semantic similarity values for each term in TP and TQ and then calculates the average value as gene functional similarity (Formula 10).
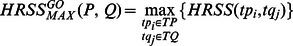
(9)

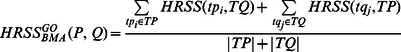
(10)where 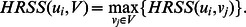


### The GO database and gene annotations

Unless stated otherwise, Gene Ontology data (released in April 2012), and gene annotation datasets (released in April 2012) for yeast and human downloaded from the Gene Ontology database [1] were used. The GO contains 22,506 BP, 2980 CC and 9341 MF terms. 88,052 annotations (45,078 without IEA annotations) of 6383 genes were present in the yeast genome, while 334,053 annotations (169,743 without IEA annotations) of 44,077 genes were present in the human genome.

### Protein-protein interaction datasets

To evaluate the performance of our new metric against other semantic similarity methods on capturing physical protein-protein interactions, following the approach of Jain and Bader [23], we built both gold standard positive (interacting) and negative (non-interacting) datasets for human and yeast genomes.

The gold standard positive dataset for human involving 2403 high quality physical/direct interactions among 1790 proteins was obtained from a core subset of the Database of Interacting Proteins (DIP) [35] (released on February 28, 2012). The DIP stores experimentally determined protein-protein interactions from a variety of sources. Six independent positive subsets for three ontologies, both including and excluding IEA annotations, were generated by selecting interactions with both proteins annotated with terms (other than the root) in their respective ontologies (Table S1). Then, six gold standard negative datasets with equal numbers of protein pairs as those in the corresponding positive datasets were independently generated by randomly choosing annotated protein pairs in BP, CC and MF, including and excluding IEA annotations, which are absent from a combined dataset of all possible protein-protein interactions. The combined dataset for human was from iRefWeb (dated April 13, 2012) and contains 36,863 direct interactions among 9853 proteins. Then yeast gold standard positive dataset involving 4218 physical/direct interactions and 2113 proteins was retrieved from DIP. The pool of all possible interactions in yeast from iRefWeb contains 71,287 direct interactions among 5947 proteins. Similarly, the positive and negative datasets for the three ontologies were generated (Table S1).

The power of a classifier was evaluated by receiver operating characteristic (ROC) curve analysis. The ROC curve presents a trade-off between true positive rate (TPR or sensitivity) and false positive rate (FPR or 1-specificity), which are calculated as *TPR* = *TP*/(*TP*+*FN*) and *FPR* = *FP*/(*FP*+TN). The ROC curve for excellent classifiers would rise steeply starting from the bottom left corner, pass close to the top left corner where both the sensitivity and specificity are 1, and finally arrive at the top right corner. In this case, the area under the ROC curve (AUC) is the largest. AUC is calculated using the trapezoidal rule, 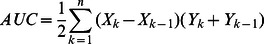, where *X_k_* is the FPR and *Y_k_* is the TPR.

### Human-mouse orthologs and randomizing simulation

Orthologous protein pairs between human and mouse were retrieved through BioMart from Ensembl (release 69) at http://www.ensembl.org/biomart/martview [36]. The annotation was not based on the reciprocal best BLAST hits but was instead based on phylogenetics. We chose only the orthologous protein pairs annotated as ‘ortholog-one2one’, both of which are present in UniProt GO annotations (released in October 2012). 5726, 5539 and 4966 human-mouse orthologs were retrieved for BP, CC and MF, respectively, after IEA annotations were excluded. The three ortholog datasets including IEA annotations were also used in this study. As a result, the functional similarity values between these orthologs on the three ontologies could be calculated using different semantic similarity measures.

To make the functional similarity comparable between different semantic similarity measures and ontologies, a Z-score analysis was applied to these one-to-one orthologs for each measure on each ontology. Since the distribution of the orthologous protein pair is unknown, we estimated the significance by randomizing the simulation process. First, an average similarity value for the observed orthologous pairs (*ASV_observed_*) was calculated. Next, an orthologous pair dataset with the same size as the observed dataset was generated by randomly choosing a mouse ortholog for each human protein without replacement, and an *ASV_random_* value was calculated on this randomized dataset. This process was repeated 1000 times (on average 1.6 protein pairs in a randomized dataset are true), and a mean and standard deviation (*stdev.*) of the 1000 *ASV_random_* were calculated. Finally, a Z-score value, defined as 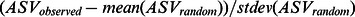, was calculated. As a result, the larger the Z-score for a semantic similarity measure on an ontology is, the less probable it is that the functional similarity measured by this method is due to chance.

### CESSM evaluation

CESSM is an online tool for evaluating GO-based semantic similarity measures using Pearson's correlation with sequence, Pfam domain and ECC similarities [34]. 13,430 protein pairs among 1039 well known proteins from multiple species and UniProt GO annotations (released on January 15, 2010) were downloaded from CESSM online. According to the notice on the CESSM website, the GO database released in January 2011 was downloaded from the GO website for the evaluation. We calculated different semantic similarity scores between the protein pairs, and then we obtained Pearson's correlations between these semantic similarity scores and sequence/Pfam/ECC similarities by running the CESSM online tool.

## Results

### Scoring physical protein-protein interactions

GO-based semantic similarity has been recognized as one of the strongest indicators for scoring and predicting protein-protein interactions, based on the following two observations: two proteins acting in the same biological process are more likely to interact than proteins involved in different processes [9]; and to interact physically, proteins should exist in close proximity, at least transiently, which suggests that colocalization may serves as a useful predictor for protein interactions [37]. In the previous work, RSS method was successfully applied to the prediction of genome-scale protein-protein interactions in yeast by combining the maximum RSS values of all term pairs associated with any two proteins for the BP and CC ontologies [4], [38]. Jain and Bader introduced a novel method, TCSS, and found that TCSS and Resnik showed the best performance in the ROC analysis and that TCSS performed better than Resnik in finding true positive interactions using the F1-measure improvement test [23].

To evaluate the performance of our improved method on capturing interacting protein pairs, the positive and negative protein-protein interaction datasets in the human and yeast genomes were generated (see Materials and Methods). HRSS were compared with our edge-based method RSS, four node-based methods TCSS, Resnik, Jiang and Lin using MAX and BMA strategies (TCSS only uses MAX), and two groupwise measures simUI and simGIC (see Section S2 in Text S1 for the implementation and definition of these methods). These methods were also compared in the following evaluation systems. The AUC values for BP and CC ontologies are much higher than those for MF under all conditions (Table 1 for human, Table S2 for yeast). This result indicated that BP and CC are better platforms than MF for predicting protein-protein interactions, supporting the two observations in the previous paragraph. Consequently, the ROC curve of MF was not shown in Figures S1 and S2. Consistent with the conclusion in [23], the similarity methods using the MAX strategy generally achieved higher AUC values than those using the BMA strategy, except for Jiang and Lin which produced the opposite result. HRSS (MAX) performed the best in CC ontology, and the same as TCSS or Resnik in BP ontology under all conditions. GO annotations, both including and excluding IEA annotations, were considered, and similar results were obtained under the two conditions. Overall, the best measure for scoring interacting protein pairs is HRSS using the maximum strategy for BP and CC ontologies.

**Table 1:** Area under the ROC curves (AUCs) for the human PPI dataset.

		Including IEA			Excluding IEA		
		BP	CC	MF	BP	CC	MF
HRSS	MAX	**0.91**	**0.84**	**0.78**	**0.90**	**0.85**	0.78
	BMA	0.90	0.83	0.77	0.89	0.83	0.76
TCSS	MAX	0.90	0.83	0.77	**0.90**	0.84	0.77
Resnik	MAX	**0.91**	0.83	**0.78**	**0.90**	0.84	0.77
	BMA	0.89	0.82	**0.78**	0.88	0.81	0.75
RSS	MAX	0.90	0.80	0.76	0.89	0.80	**0.80**
	BMA	0.87	0.75	0.75	0.87	0.74	0.77
simGIC		0.88	0.76	0.73	0.87	0.75	0.73
simUI		0.86	0.73	0.69	0.85	0.72	0.70
Lin	MAX	0.80	0.67	0.71	0.83	0.69	0.75
	BMA	0.86	0.73	0.77	0.86	0.71	0.77
Jiang	MAX	0.80	0.67	0.70	0.82	0.70	0.75
	BMA	0.84	0.71	0.77	0.83	0.69	0.76

The tests were carried out separately for BP and CC ontologies. The maximum (MAX) and best-match average (BMA) strategies were used for datasets including or excluding the IEA (Inferred from Electronic Annotation) evidence code. For each group, the top scores are in bold.

### Choosing reasonable thresholds for positive and negative protein-protein interactions

Both high-quality positive and negative datasets of protein-protein interactions are required for comparative statistical analyses including learning and validation processes [39]. However, determining reasonable HRSS cutoff values for high-quality positive and negative protein-protein interactions was a challenge. In addition to FPR and TPR, another three measures, PPV, NPV and the Youden index [40], were considered. The ROC curve for yeast in BP ontology including IEA annotation was taken as an example to follow for the selection of thresholds.

**Figure 2:**
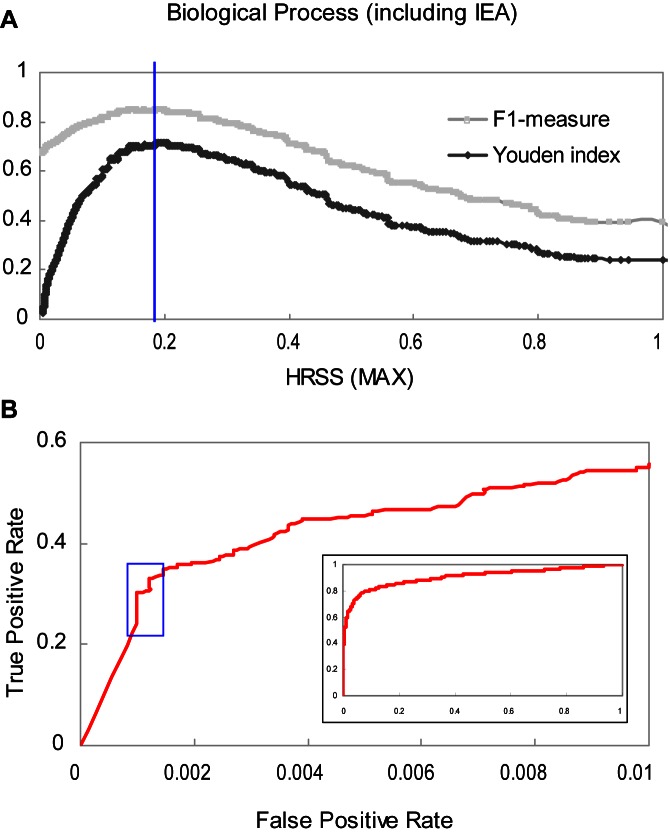
An illustration of choosing the HRSS (MAX) thresholds for positive and negative protein-protein interactions. The ROC curve for yeast in BP ontology including IEA annotations was taken as an example. (**A**) The distribution of the F1-measure and Youden index with different HRSS values from 0 to 1. The Youden index was used to choose an HRSS cutoff (the peak indicated by the blue line) that was able to roughly differentiate between positive and negative protein interactions. Both the F1-measure and Youden index reach their maximum values when HRSS is approximately 0.2 in this example. (**B**) The ROC curve provides one way to choose the HRSS cutoff for selecting high-quality positive interactions. The ‘steeply rising line’ (within the blue box) sharply increases sensitivity (TPR) but maintains a low false positive rate (FPR) at the same time. The point near the end of the ‘steeply rising line’ has a low false positive rate of predicted protein interactions.

The Youden index, *Y* =  *Specificity*+*Sensitivity*-*1*, provides a criterion for choosing the cutoff to maximize the sum of specificity and sensitivity in an ROC curve. The maximum (0.72) of the Youden index was achieved when the HRSS cutoff value was close to 0.2. We also tested the F1-measure, *F =  2(PPV×TPR)/(PPV+TPR)* where *PPV =  TP/(TP+FP)*, and achieved a similar threshold (Figure 2A). Moreover, through the distributions of protein-protein interactions from gold standard positive and negative datasets falling in various HRSS values (Figure S3), we found that the most (93% for yeast in BP ontology) of negative interactions fall into an HRSS lower than 0.2, whereas 78% of positive interactions have an HRSS higher than 0.2. As a result, the protein pairs with an HRSS higher than 0.2 are likely to interact with each other and those lower than 0.2 likely do not. However, using a single Youden index is not generally recommended because there is not a single best cutoff derived from ROC curves [41]. Therefore, different measures should be considered to yield both high-quality positive and negative datasets.

The ROC curve for excellent classifiers would rise steeply starting from the bottom left corner and pass close to the top left corner where both the sensitivity and specificity are 1. The ‘steeply rising line’ (within the blue box in Figure 2B) sharply increases sensitivity (TPR, the fraction of true positives in positives) but still maintains a low false positive rate (FPR, the fraction of false positives in negatives) at the same time. PPV, the proportion of predicted positives that are true, provides a way for measuring the accuracy of an algorithm. We aimed for the predicted positive interactions to have a high accuracy and low false positive rate (close to the end of the ‘steeply rising line’). A reasonable threshold for high-confidence interactions was 0.7 because it corresponded to an accuracy (PPV) of 0.996, a false positive rate of only 0.001 and coverage on observed positives (TPR) of 0.32 (Table S3). Additionally, we aimed for the predicted negative interactions to have a high prediction accuracy (NPV, *NPV =  TN/(TN+FN)*, the proportion of predicted negatives that are true) and reasonable coverage on observed negatives (specificity, 1-FPR). The cutoff for this prediction of high-quality negatives was 0.1, corresponding to a prediction accuracy of 0.859 and specificity of 0.69 (Table S3).

Based on the three cutoffs (0.2 for the Youden index, and 0.7 and 0.1 for the other indexes) chosen above, the HRSS (MAX) values for the BP ontology in yeast, using all GO annotations, were divided into four groups with high confidence (H, 0.7≤HRSS≤1), medium-high confidence (M_H_, 0.2≤HRSS<0.7), medium-low confidence (M_L_, 0.1≤HRSS<0.2) and low confidence (L, 0≤HRSS<0.1) (Table 2). The HRSS values of CC ontology in yeast could also be split to four groups: high confidence (H, 0.5≤HRSS≤1), medium-high confidence (M_H_, 0.2≤HRSS<0.5), medium-low confidence (M_L_, 0.1≤HRSS<0.2) and low confidence (L, 0≤HRSS<0.1) (Table 2, Table S3). Similar HRSS thresholds were chosen for human (Table 2, Table S4).

**Table 2:** Four groups of HRSS values with different confidences for positive and negative protein-protein interactions in BP and CC ontologies.

Species	GO	L	M_L_	M_H_	H
Yeast	BP	[0, **0.1**)	[0.1, **0.2**)	[0.2, 0.7)	[**0.7**, 1]
	CC	[0, **0.1**)	[0.1, **0.2**)	[0.2, 0.5)	[**0.5**, 1]
Human	BP	[0, **0.1**)	[0.1, **0.2**)	[0.2, 0.6)	[**0.6**, 1]
	CC	[0, **0.1**)	[0.1, **0.2**)	[0.2, 0.5)	[**0.5**, 1]

An ROC analysis showed that the HRSS method using the maximum strategy on GO annotations including IEA code performed best among all methods on distinguishing true protein interactions from false interactions. Since no one ‘best’ threshold could be obtained directly from the ROC analysis, five indexes, TPR, FPR, PPV, NPV and the Youden index, were used to determine the reasonable HRSS cutoffs (in bold) for selecting positive and negative interactions. Accordingly, the HRSS values were divided into four groups with low (L), medium-low (M_L_), medium-high (M_H_) and high (H) confidences.

### Assessing functional similarity between human-mouse orthologs

**Figure 3:**
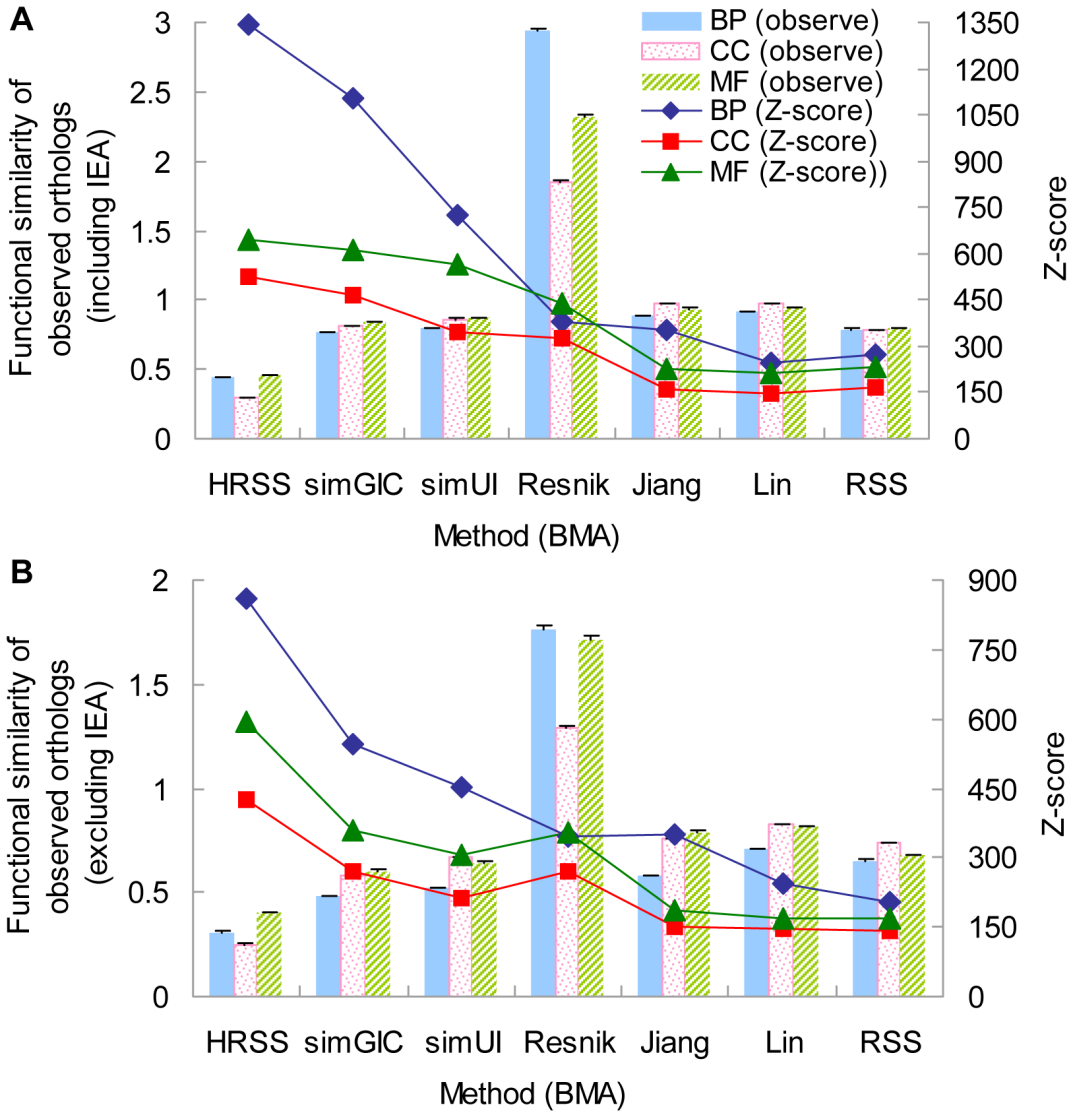
Statistical significance of system for evaluating the functional similarity (BMA) of human-mouse orthologs. The evaluation system was based for the BP, CC and MF ontologies (**A**) including or (**B**) excluding IEA annotations. The histograms (measured on the left y-axis) indicate the mean and standard error of the functional similarities of observed orthologs, and the lines (measured on the right y-axis) show the Z-score values calculated from the ASVs of observed orthologous pairs and randomized pairs (see Materials and Methods). HRSS achieved the highest Z-score in all cases. Error bars indicate one standard error.

In general, the notion that one-to-one orthologs are functionally similar holds well, although there are notable cases of major differences in the functions of orthologs at greater evolutionary distances, particularly across the primary kingdom divides [42]. Mouse is commonly used as a model organism for studying human biology and disease [43]. Therefore, we investigated the performance of different semantic similarity methods on the degree of functional conservation in human-mouse one-to-one orthologs (see Materials and Methods). Resnik showed the highest functional similarity of the ‘true’ orthologos in all datasets because it alone does not produce normalized scores. HRSS with BMA showed a fairly low functional similarity (histograms in Figure 3, Table S5), whereas HRSS with MAX produced a functional similarity similar to that of simGIC, simUI and TCSS (histograms in Figure S4, Table S5).

To make the functional similarities comparable between different semantic similarity measures and ontologies, a Z-score analysis was applied to these one-to-one orthologs for each measure in each ontology (see Materials and Methods). When BMA was used, HRSS showed the best performance consistently (lines in Figure 3, Table S6), followed by the groupwise method, simGIC. The ASV of HRSS (BMA) for observed orthologs was at least 861 (BP), 592 (MF) or 423 (CC) standard deviations higher than the mean values for randomized orthologs. When MAX was used, simGIC was best for all three ontologies considering all GO annotations (lines in Figure S4A, Table S7), whereas HRSS (MAX) was best for MF and CC ontologies after the IEA annotations have been removed (lines in Figure S4B, Table S7).

BMA and MAX were also compared in evaluating the functional similarity between orthologs. As listed in Table 3, BMA performed better than MAX for all pairwise methods. For example, HRSS (BMA) achieved Z-score that was more than 136 (including IEA) or 47 (excluding IEA) higher than HRSS (MAX) obtained. Consequently, according to the better performance of the BMA approach, HRSS was statistically most suitable for evaluating the functional similarity between human-mouse orthologs.

**Table 3:** Comparison of two pairwise strategies, MAX and BMA, on estimating the functional conservation between human-mouse orthologs using Z-score analysis.

	Including IEA			Excluding IEA		
	BP	CC	MF	BP	CC	MF
HRSS	815.28	136.27	345.35	329.13	47.26	180.37
Resnik	94.43	57.75	67.31	51.75	6.03	37.55
Jiang	250.30	105.12	145.45	196.93	55.78	62.00
Lin	160.04	92.19	124.88	106.04	50.85	49.37
RSS	99.84	52.58	93.95	42.95	22.01	37.65

The difference between the two approaches was measured by the Z-score of the BMA approach minus the Z-score of MAX. This table showed a higher functional conservation of human-mouse orthologs statistically using the BMA approach.

### Estimating correlation with sequence, Pfam and Enzyme Commission Class similarities

**Figure 4:**
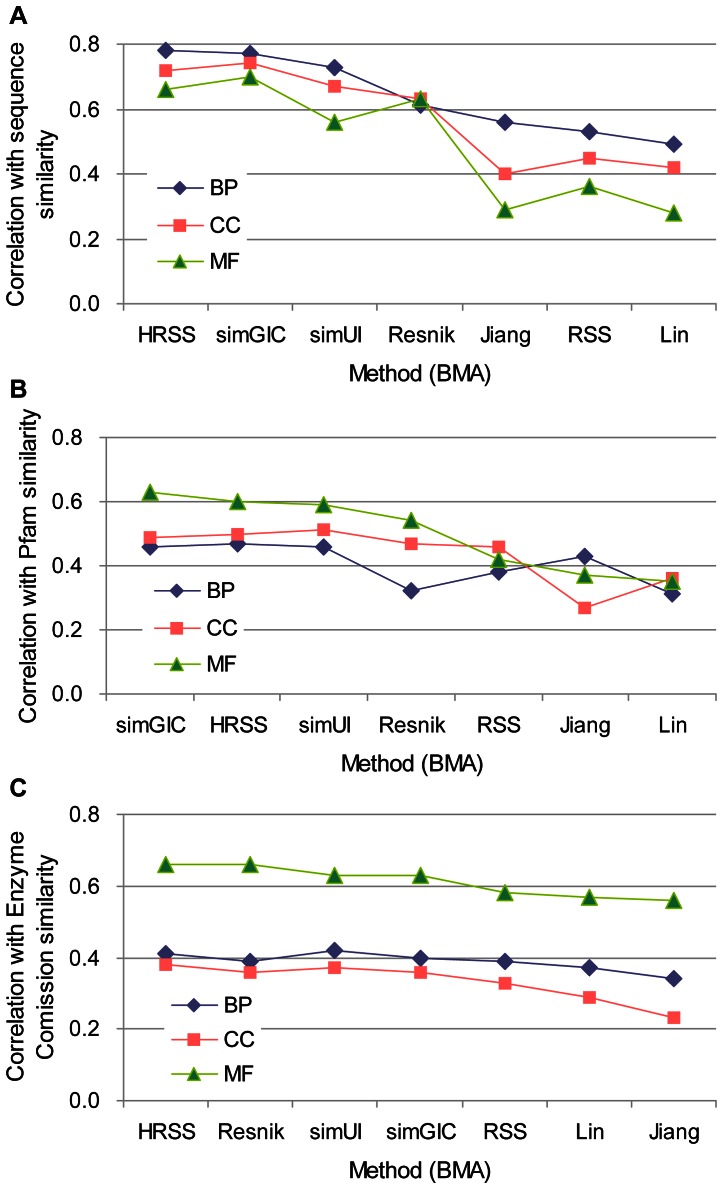
Correlation between semantic similarity (BMA) and the CESSM dataset (including IEA). CESSM displays the data of (**A**) sequence, (**B**) Pfam and (**C**) ECC similarities. The evaluation was carried out for UniProt protein pairs from the CESSM database in the BP, CC and MF ontologies.

**Figure 5:**
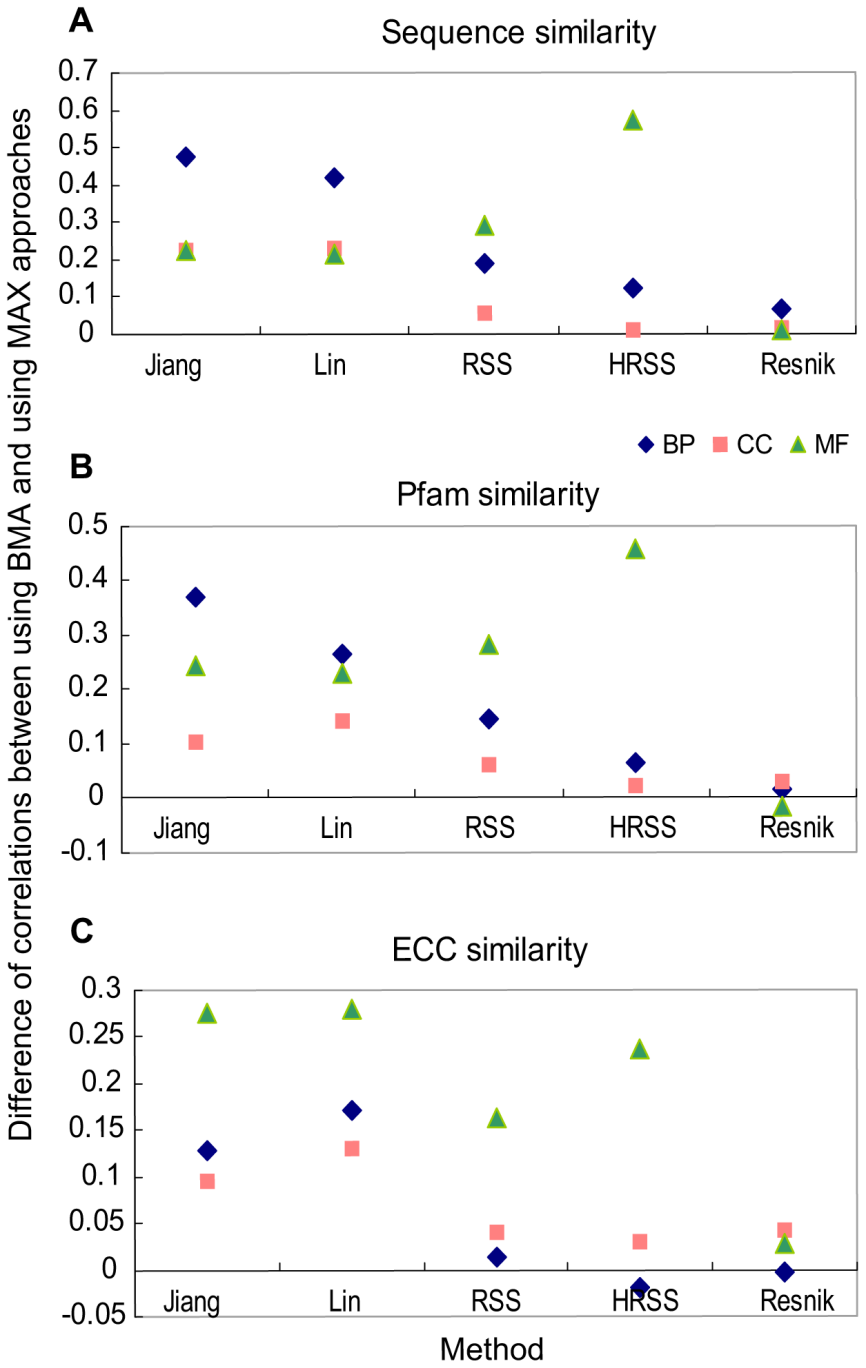
Comparison of two pairwise strategies, BMA and MAX, on correlation with CESSM dataset (including IEA). The CESSM dataset shows the similarity of (**A**) sequence, (**B**) Pfam and (**C**) ECC for UniProt protein pairs. The difference between the two strategies was measured by the correlation coefficient of the BMA strategy minus that of MAX.

The evaluation of 13,430 protein pairs using UniProt GO annotations in all three ontologies was performed based on the CESSM platform (see Materials and Methods). The BMA was applied to the pairwise methods (Figure 4 and Figure S5). Both HRSS and simGIC outperformed the others in sequence similarity whether IEA annotations were included or not (with a correlation of approximately 0.8 in BP). HRSS, simGIC and simUI performed better in Pfam similarity than the others (with a correlation of approximately 0.6 in MF). The three aforementioned methods and Resnik showed a similar correlation for ECC (0.6 in MF, and 0.4 in BP and CC). We also tested the MAX strategy. HRSS (MAX) showed a poor correlation when IEA annotations were considered (Figure S6A-C), but was comparable to the others when IEA annotations were discarded (Figure S6D-F). Similar to the evaluation of human-mouse orthologs, the two pairwise rules, BMA and MAX, were compared with each other. As shown in Figure 5 and Figure S7, the methods using BMA generally obtained higher correlations. For example, using HRSS with the MF ontology (including IEA), the correlation with sequence similarity increased from 0.09 with MAX to 0.66 with BMA, and the correlation with Pfam similarity increased by 0.46 when BMA was used.

In summary, BMA was more suitable than MAX for the CESSM platform. Accordingly, HRSS (BMA) and simGIC were best for correlating with sequence similarity and Pfam similarity, whereas each of the seven methods gave similar results with the ECC dataset.

## Discussion

We tested the performance of GO-based semantic similarity measures on the correlation with gene expression using microarray data from yeast and human. Both Pearson's and Spearman's correlation coefficients between semantic similarity and gene co-expression were calculated, but poor correlations were obtained (Figure S8, see Section 3 in Text S1 for detail). To ascertain the underlying data trends, the expression similarity values had been transformed by several means. Sevilla et al. averaged the expression similarity values over uniform semantic similarity intervals [29]. The averaging procedure dramatically improved correlation coefficients for the Resnik similarity measure but not for the Jiang and Lin measures, though the relationship was not strictly linear. Jain and Bader calculated average gene expression from Pearson's correlation using Figher's z transformation, and found that TCSS rather than Resnik produced the highest correlation [23]. Poor correlations were obtained using the ‘raw’ Pearson's correlation values. However, the relationship between gene expression and GO annotation may be inflated by the averaging procedures, which indicates that the correlations between GO semantic similarity and gene co-expression are sensitive to the data and procedures used.

Two commonly used pairwise strategies, the maximum and best-match average, were chosen based on node- and edge-based semantic similarity methods to evaluate the functional similarity between two gene products. In general, the BMA approach was a better choice than MAX for estimating the functional conservation of orthologs (Table 3), and evaluating correlations with sequence, domain and ECC similarities (Figure 5, Figure S7). The MAX strategy which considers the best match among all term pairs of two gene products, could be potentially adversely affected by incorrect annotations or the noise along with the IEA annotations [33]. This may explain why HRSS (MAX) showed poor and sometimes unstable performance including IEA annotations in the evaluation based on human-mouse orthologs (Figure S4A and Table S7) and CESSM platform (Figure S6A and B in MF ontology). The MAX strategy could assess if two gene products share a similar function, but underestimates their dissimilarity. As a result, the MAX approach is not suitable for assessing the global functional similarity of two gene products. The BMA strategy considered all term pairs of two gene products but only the best matching for each gene product. Overall, this strategy is better than MAX [11]. However, the conclusion was reversed when predicting positive protein-protein interactions (Table 1, Table S2), consistent with the results of Jain and Bader [23]. The conclusion is reasonable because two physically interacting proteins need to participate in a similar biological process (share similar or identical BP terms) and to locate in a same cellular component (share similar or identical CC terms) only once.

GO annotations were grouped into two main classes. Some were assigned by expert curators based on experimental evidences or other evidences such as sequence similarity, structural similarity and genomic context, and others were inferred electronically (IEA) without individual curator supervision. A large portion of gene annotations fall into the electronically inferred class. In our study, IEA annotations comprised 49% of the GO annotations in yeast and human, and the percentage in the UniProt GO annotation database (released in October 2012) was almost 99% of 110,275,783 total annotations. Interestingly, the percentages of proteins that have only IEA annotations vary among different annotation corpora, with zero occurring in yeast, 65% in human and 99% in UniProt. IEA annotations were widely considered unreliable, so they were assigned a lower weight or were disregarded in many studies. A recent study comprehensively evaluated the quality of the IEA annotations based on UniProt GO annotations in terms of reliability, coverage and specificity measures [44]. It concluded that the electronic annotations were significantly improved in recent years, and that the reliability of electronic annotations now rivals that of non-experimentally curated annotations. We have consistently observed that the addition of IEA annotations either led to a higher Z-score for statistically estimating the functional similarity between human-mouse orthologs (using the BMA pairwise strategy) (Tables S6), or did not change the results of both correlating with the CESSM dataset (using BMA) (Figure 4 and Figures S5) and inferring protein-protein interactions (using MAX) (Table 1 and Table S2). Therefore, IEA annotations are useful in general, especially for large-scale studies as suggested by Guzzi et al. [33].

There are two major caveats of our method. First, the IC of a term is normally calculated from the annotation information, which is the number of genes annotated with the term and its descendants in a corpus, and is thus referred to as a ‘corpus-based’ metric. An alternative approach to estimate the IC of GO terms is based on the GO structure itself, such as those in Teng et al. and Seco et al. [27], [45], although this type of approach is less commonly used. The semantic similarity methods that use the corpus-based metric have the following inherent limitations: (i) Using the corpus-based IC risks bias towards the topics that have been more thoroughly studied. The annotation information of a term in a particular corpus is dependent on the studies of the annotated genes. Therefore, in some cases IC does not reflect the biological specificity of the term [33]. This phenomenon of corpus bias cannot be resolved. (ii) There are a number of terms (29%, 30% and 26% of GO terms in BP, CC and MF ontologies, respectively, in UniProt annotation corpus released in October 2012) and their descendants that have no annotation information. The IC of these terms cannot be obtained, leading to an issue of calculating semantic similarity scores involving the unannotated terms. This problem has no influence on calculating the semantic similarity between pairs of genes in an annotation corpus, however. (iii) The IC score for the same term changes as the knowledge in a specific annotation corpus changes over time and also varies across different annotation corpora. Therefore, the semantic similarity scores between term pairs or gene pairs need to be constantly updated.

Second, only a single common ancestor, the MICA, was used in our methods. In fact, GO terms can have several disjunctive common ancestors (DCAs), all of which contribute to various degrees to the semantic similarity of the terms. To effectively explore the multiple inheritance relationships present in GO, Couto et al. designed the GraSM approach, which replaces the IC of the MICA with the average IC of all DCAs [21], and recently proposed DiShIn [22] to update GraSM. The shared IC obtained through DiShIn was shown to improve the correlation of the semantic similarity measures with sequence similarity [22]. It will be useful to adopt DiShIn to HRSS in the future by replacing the MICA with DiShIn and by modifying the definition of component *γ* as the average or maximum local distance between the two terms and their DCAs.

In summary, we presented a new GO-based semantic similarity method, HRSS, which is an improved version of the edge-based measure RSS [4]. Based on the framework of the RSS algorithm, we adopted the concept of information content and updated two components (*α* and *β*) of RSS (Figure 1A). Thus, like RSS, HRSS not only considered the specificity of the most informative common ancestor of the two relevant terms (component *α_IC_*), but also comprehensively included the generality of the two terms (component *β_IC_*) and their local similarity relative to the most informative ancestor (converted from component *γ*) (Figure 1B). Different performances for semantic similarity measures have been reported under different circumstances: Resnik, simGIC and TCSS are often identified as the best measures [33]. In our study, the performance of HRSS on different biological issues was assessed independently against other methods. HRSS (BMA) was the most suitable measure for evaluating the functional conservation of human-mouse orthologs. For inferring high-quality positive and negative protein-protein interactions, HRSS was slightly superior to Resnik and TCSS in CC ontology and performed similarly to them in BP ontology. Using the CESSM platform, both HRSS (BMA) and simGIC were best at correlating with sequence similarity and Pfam similarity, and all of the methods gave nearly similar results for correlating with ECC similarity. RSS, Jiang and Lin performed poorly in general. As an edge-based measure, RSS relies only on the intrinsic structure of the GO DAG, causing some problems (see Introduction). Jiang and Lin only assess the two terms with interest relative to their common ancestor, and therefore neglect the specificity of the terms in the GO DAG. Furthermore, we used several indexes (the Youden index, TPR, FPR, PPV and NPV) of the ROC plots to simply choose the thresholds for high-quality positive and negative protein-protein interactions. Based on the thresholds, the HRSS values were divided into four groups with high confidence (H), medium-high confidence (M_H_), medium-low confidence (M_L_) and low confidence (L) (Table 2). Although the method to define the thresholds is not completely objective, we hope the thresholds chosen are useful for predicting high-quality positive and negative protein-protein interactions or assessing the confidence of interactions using HRSS.

## Supporting Information

Figure S1 **ROC curves comparing different methods based on the human protein-protein interaction datasets (including IEA).** The evaluation was carried out for the BP and CC ontologies. The (**A** and **B**) maximum (MAX) and (**C** and **D**) best-match average (BMA) pairwise rules were used in the ROC analysis.
(PDF)

Figure S2 **ROC curves comparing different semantic similarity methods based on yeast protein-protein interaction datasets (including IEA).** The evaluation was done for the BP and CC ontologies. The (**A** and **B**) MAX and (**C** and **D**) BMA pairwise rules were applied.
(PDF)

Figure S3 **Distributions of the positive and negative interacting protein pairs with various HRSS (MAX) values.** HRSS was calculated for (**A** and **B**) yeast and (**C** and **D**) human protein-protein interactions in the gold standard positive and negative datasets for BP and CC ontologies (including IEA annotations). The [0,1] interval of HRSS values is equally divided into 11 categories, which are {[0.1×*i*, 0.1×(*i*+1)), *i* = 0,1,…,9} plus HRSS = 1.
(PDF)

Figure S4 **Statistical significance of system for evaluating the functional similarity (MAX) of human-mouse orthologs.** The evaluation system was based on BP, CC and MF ontologies (**A**) including or (**B**) excluding IEA annotations. The histograms (measured on the left y-axis) indicate the mean and standard error of the functional similarities of observed orthologs, and the lines (measured on the right y-axis) show the Z-score values calculated from the ASVs of observed orthologous pairs and randomized pairs (see Materials and Methods). Error bars indicate one standard error.
(PDF)

Figure S5 **Correlation between semantic similarity (BMA) and the CESSM dataset (excluding IEA).** CESSM displays the data of (**A**) sequence, (**B**) Pfam and (**C**) ECC similarities.
(PDF)

Figure S6 **Correlation between semantic similarity (MAX) and the CESSM dataset (including and excluding IEA).** CESSM holds the data of (**A** and **D**) sequence, (**B** and **E**) Pfam and (**C** and **F**) ECC similarities.
(PDF)

Figure S7 **Comparison of two pairwise strategies, MAX and BMA, on correlation with CESSM dataset (excluding IEA)**. The CESSM dataset shows the similarity of (**A**) sequence, (**B**) Pfam and (**C**) ECC for UniProt protein pairs. The difference between the two strategies was measured by the correlation coefficient of the BMA strategy minus that of MAX.
(PDF)

Figure S8 **Correlation between semantic similarity and gene expression similarity.** The (**A** and **B**) BMA and (**C** and **D**) MAX pairwise strategies were used. The evaluation based on the BP and MF ontologies (including IEA) was carried out for human and yeast, independently.
(PDF)

Table S1 **The sizes of gold standard positive and negative interaction datasets used for ROC analysis in human and yeast.**
(PDF)

Table S2 **Area under the ROC curves (AUCs) for the yeast PPI dataset.**
(PDF)

Table S3 **Indexes used for evaluating the performance of HRSS (MAX) on scoring protein-protein interactions in yeast.**
(PDF)

Table S4 **Indexes used for evaluating the performance of HRSS (MAX) on scoring protein-protein interactions in human.**
(PDF)

Table S5 **Summary of the functional similarities of observed human-mouse orthologs calculated by various semantic similarity methods (including IEA).**
(PDF)

Table S6 **Z-score analysis of various semantic similarity methods (BMA) for estimating the functional similarity among human-mouse orthologs.**
(PDF)

Table S7 **Z-score analysis of various semantic similarity methods (MAX) for estimating the functional similarity among human-mouse orthologs.**
(PDF)

Text S1 **Describes additional details of the semantic similarity methods used in this study, as well as additional information regarding the correlation between semantic similarities and gene co-expression.**
(PDF)
