# Supplementary material for: Improving the Measurement of Semantic Similarity between Gene Ontology Terms and Gene Products: Insights from an Edge- and IC-Based Hybrid Method
Source: PLoS One. 2013 May 31;8(5):e66745. doi: 10.1371/journal.pone.0066745 (PMC3669204; doi:10.1371/journal.pone.0066745)
Supplement: Figure S1 — ROC curves comparing different methods based on the human protein-protein interaction datasets (including IEA). The evaluation was carried out for the BP and CC ontologies. The (A and B) maximum (MAX) and (C and D) best-match average (BMA) pairwise rules were used in the ROC analysis. (PDF) [file pone.0066745.s001.pdf]

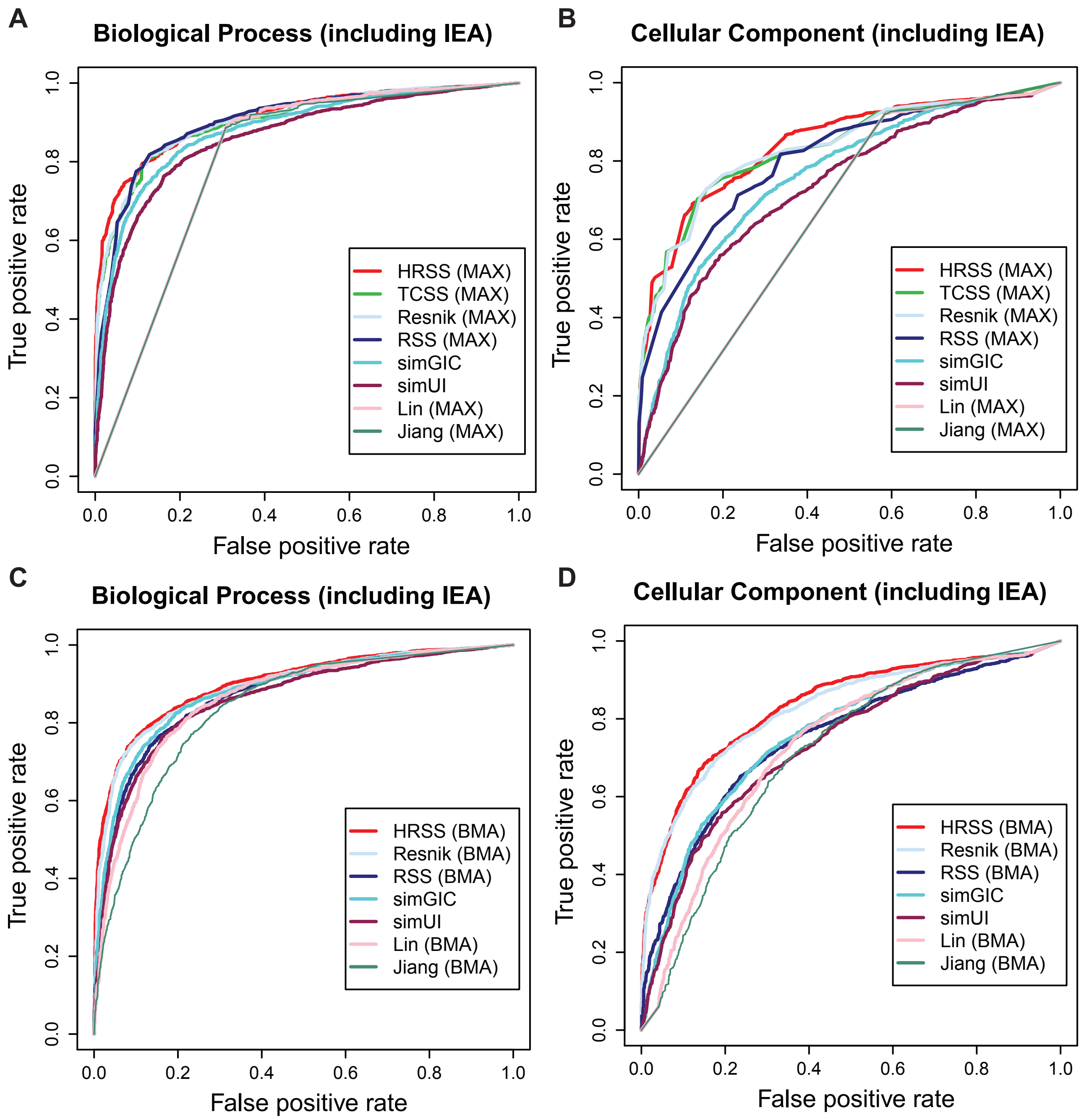

Figure S1. ROC curves comparing different methods based on the human protein-protein interaction datasets (including IEA). The evaluation was carried out for the BP and CC ontologies. The (**A** and **B**) maximum (MAX) and (**C** and **D**) best-match average (BMA) pairwise rules were used in the ROC analysis.
