# Supplementary material for: Improving the Measurement of Semantic Similarity between Gene Ontology Terms and Gene Products: Insights from an Edge- and IC-Based Hybrid Method
Source: PLoS One. 2013 May 31;8(5):e66745. doi: 10.1371/journal.pone.0066745 (PMC3669204; doi:10.1371/journal.pone.0066745)
Supplement: Figure S2 — ROC curves comparing different semantic similarity methods based on yeast protein-protein interaction datasets (including IEA). The evaluation was done for the BP and CC ontologies. The (A and B) MAX and (C and D) BMA pairwise rules were applied. (PDF) [file pone.0066745.s002.pdf]

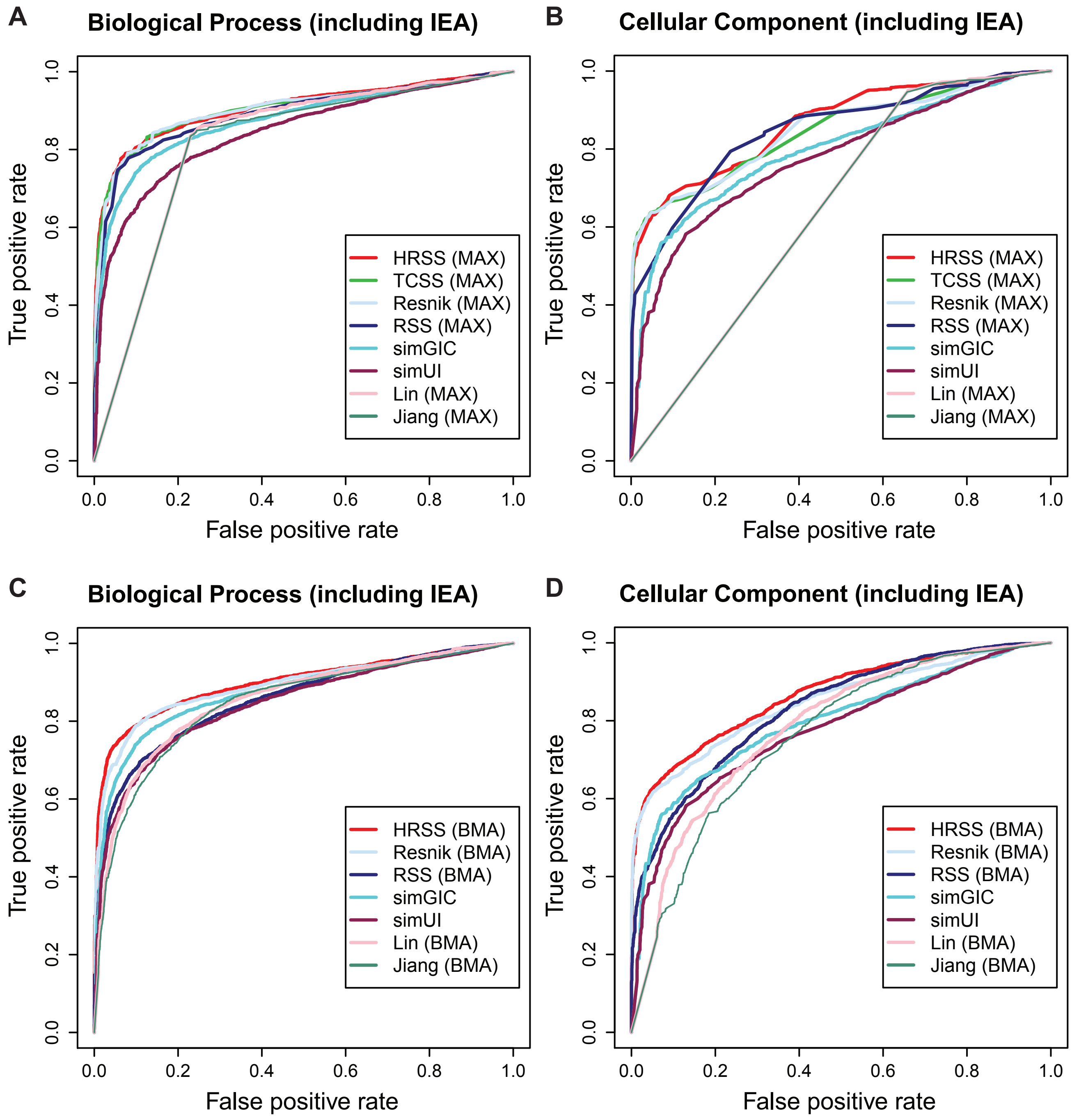

Figure S2. ROC curves comparing different semantic similarity methods based on yeast protein-protein interaction datasets (including IEA). The evaluation was done for the BP and CC ontologies. The (**A** and **B**) MAX and (**C** and **D**) BMA pairwise rules were applied.
