# Supplementary material for: Improving the Measurement of Semantic Similarity between Gene Ontology Terms and Gene Products: Insights from an Edge- and IC-Based Hybrid Method
Source: PLoS One. 2013 May 31;8(5):e66745. doi: 10.1371/journal.pone.0066745 (PMC3669204; doi:10.1371/journal.pone.0066745)
Supplement: Figure S3 — Distributions of the positive and negative interacting protein pairs with various HRSS (MAX) values. HRSS was calculated for (A and B) yeast and (C and D) human protein-protein interactions in the gold standard positive and negative datasets for BP and CC ontologies (including IEA annotations). The [0,1] interval of HRSS values is equally divided into 11 categories, which are {[0.1×i, 0.1×(i+1)), i = 0,1,…,9} plus HRSS = 1. (PDF) [file pone.0066745.s003.pdf]

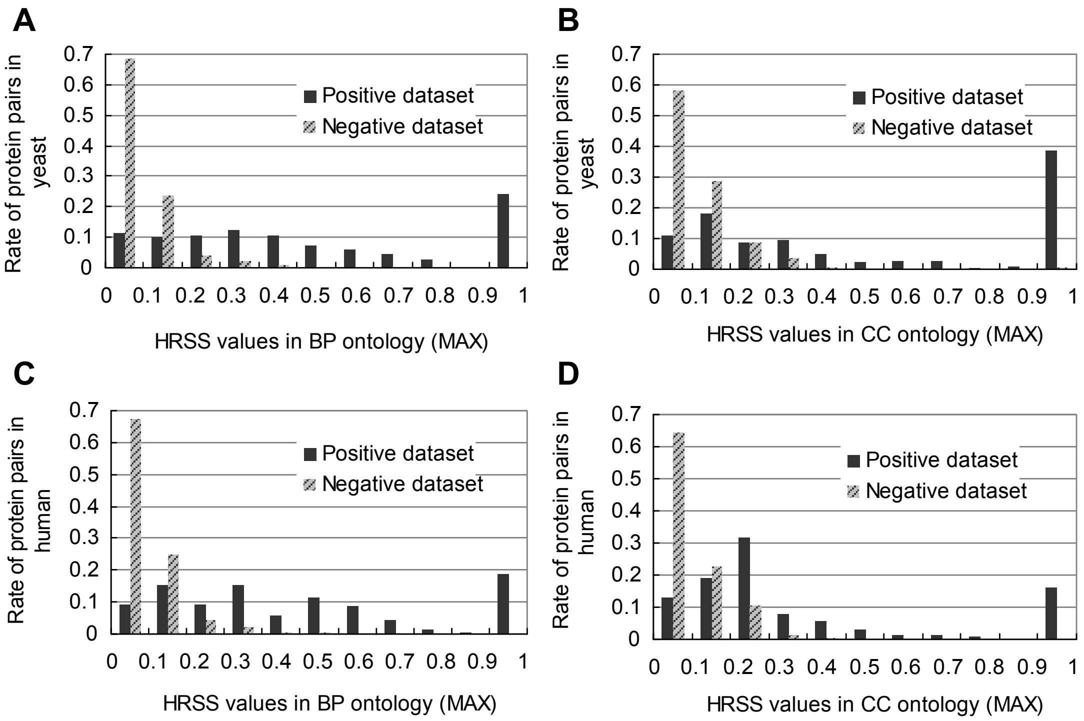

Figure S3. Distributions of the positive and negative interacting protein pairs with various HRSS (MAX) values. HRSS was calculated for (**A** and **B**) yeast and (**C** and **D**) human protein-protein interactions in the gold standard positive and negative datasets for BP and CC ontologies (including IEA annotations). The [0,1] interval of HRSS values is equally divided into 11 categories, which are $\{[0.1\times i, 0.1\times(i+1)), i = 0,1, \ldots, 9\}$ plus HRSS = 1.
