# Supplementary material for: Improving the Measurement of Semantic Similarity between Gene Ontology Terms and Gene Products: Insights from an Edge- and IC-Based Hybrid Method
Source: PLoS One. 2013 May 31;8(5):e66745. doi: 10.1371/journal.pone.0066745 (PMC3669204; doi:10.1371/journal.pone.0066745)
Supplement: Figure S4 — Statistical significance of system for evaluating the functional similarity (MAX) of human-mouse orthologs. The evaluation system was based on BP, CC and MF ontologies (A) including or (B) excluding IEA annotations. The histograms (measured on the left y-axis) indicate the mean and standard error of the functional similarities of observed orthologs, and the lines (measured on the right y-axis) show the Z-score values calculated from the ASVs of observed orthologous pairs and randomized pairs (see Materials and Methods). Error bars indicate one standard error. (PDF) [file pone.0066745.s004.pdf]

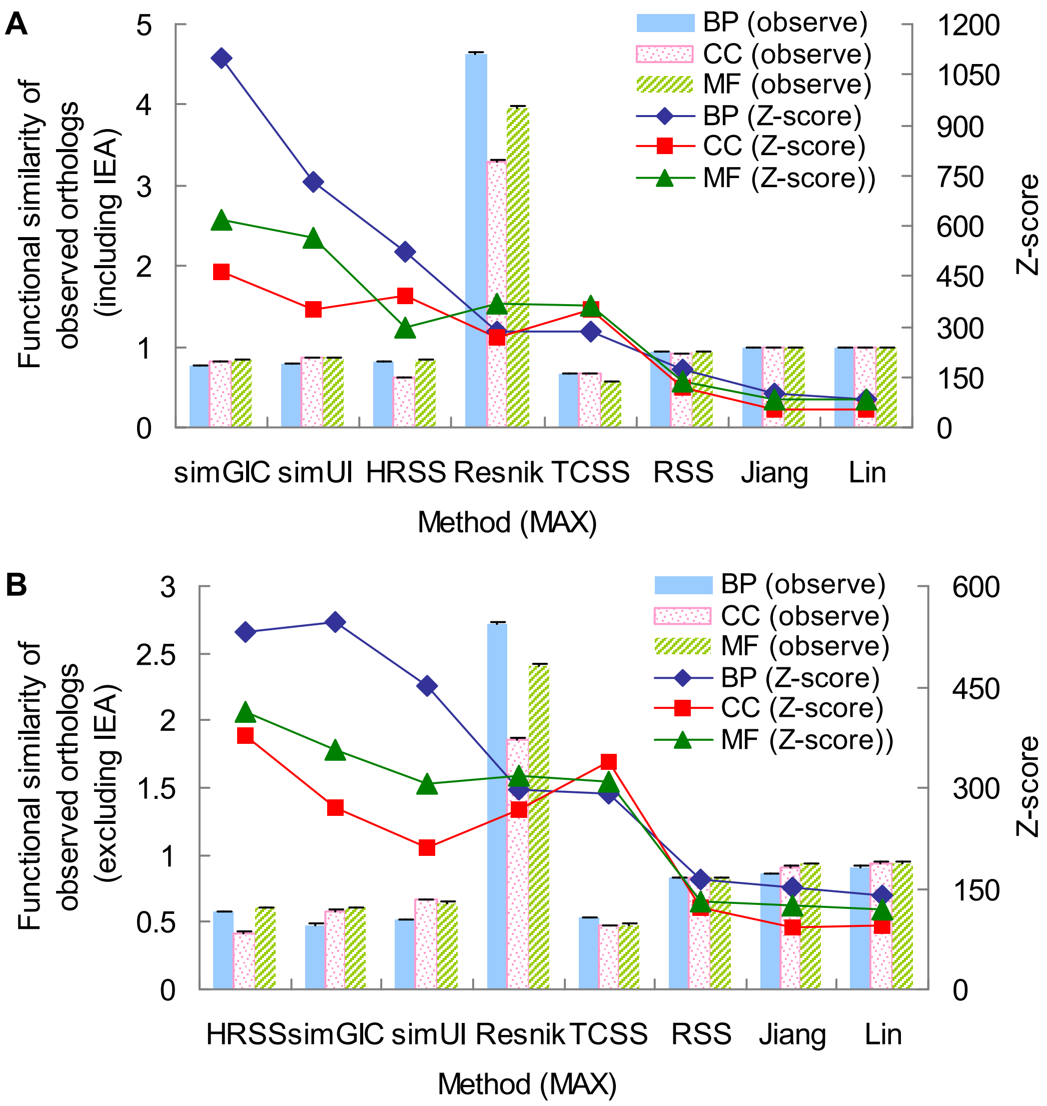

Figure S4. Statistical significance of system for evaluating the functional similarity (MAX) of human-mouse orthologs. The evaluation system was based on BP, CC and MF ontologies (**A**) including or (**B**) excluding IEA annotations. The histograms (measured on the left y-axis) indicate the mean and standard error of the functional similarities of observed orthologs, and the lines (measured on the right y-axis) show the Z-score values calculated from the ASVs of observed orthologous pairs and randomized pairs (see Materials and Methods). Error bars indicate one standard error.
