# Supplementary material for: Improving the Measurement of Semantic Similarity between Gene Ontology Terms and Gene Products: Insights from an Edge- and IC-Based Hybrid Method
Source: PLoS One. 2013 May 31;8(5):e66745. doi: 10.1371/journal.pone.0066745 (PMC3669204; doi:10.1371/journal.pone.0066745)
Supplement: Figure S5 — Correlation between semantic similarity (BMA) and the CESSM dataset (excluding IEA). CESSM displays the data of (A) sequence, (B) Pfam and (C) ECC similarities. (PDF) [file pone.0066745.s005.pdf]

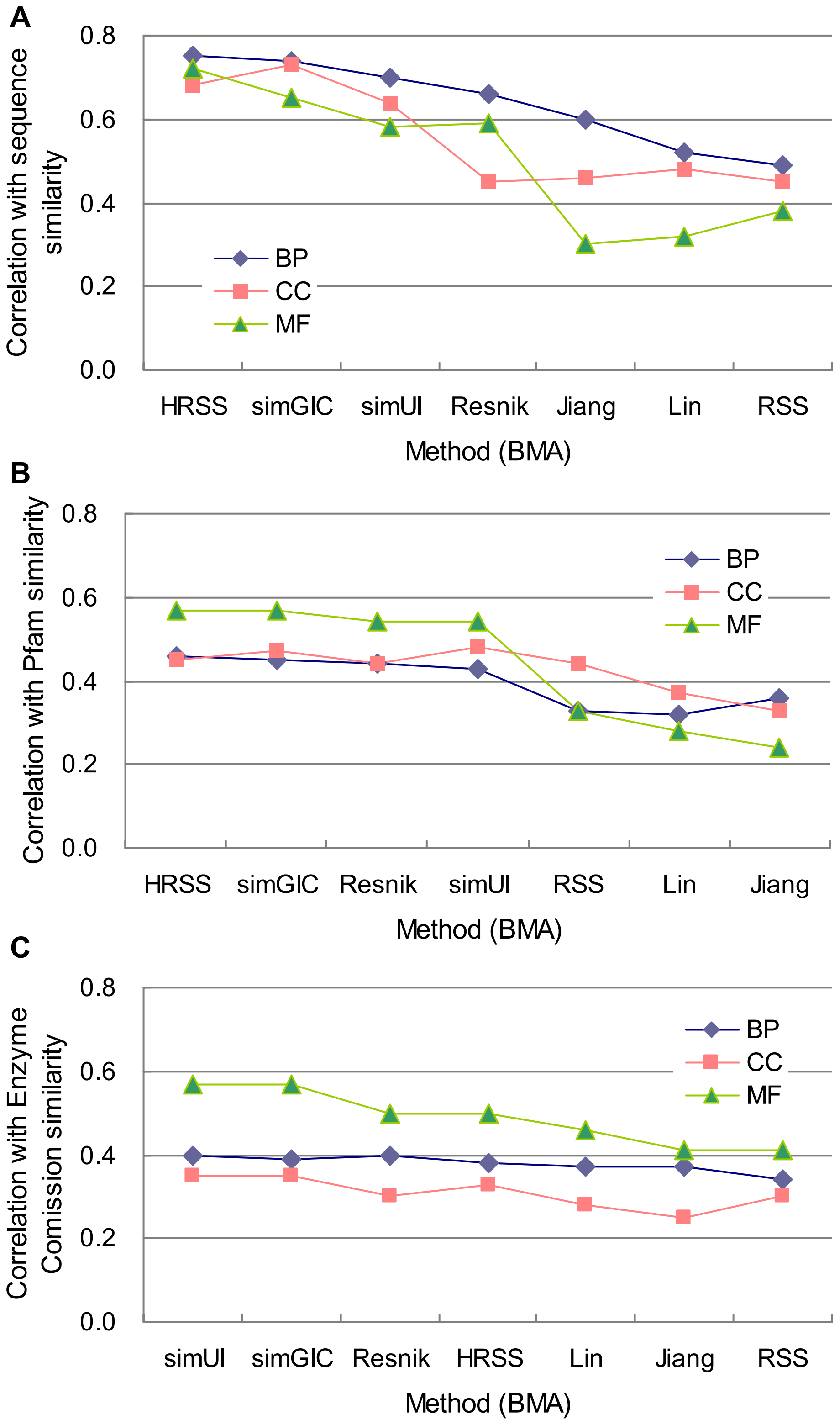

Figure S5. Correlation between semantic similarity (BMA) and the CESSM dataset (excluding IEA). CESSM displays the data of (**A**) sequence, (**B**) Pfam and (**C**) ECC similarities.
