# Supplementary material for: Improving the Measurement of Semantic Similarity between Gene Ontology Terms and Gene Products: Insights from an Edge- and IC-Based Hybrid Method
Source: PLoS One. 2013 May 31;8(5):e66745. doi: 10.1371/journal.pone.0066745 (PMC3669204; doi:10.1371/journal.pone.0066745)
Supplement: Figure S6 — Correlation between semantic similarity (MAX) and the CESSM dataset (including and excluding IEA). CESSM holds the data of (A and D) sequence, (B and E) Pfam and (C and F) ECC similarities. (PDF) [file pone.0066745.s006.pdf]

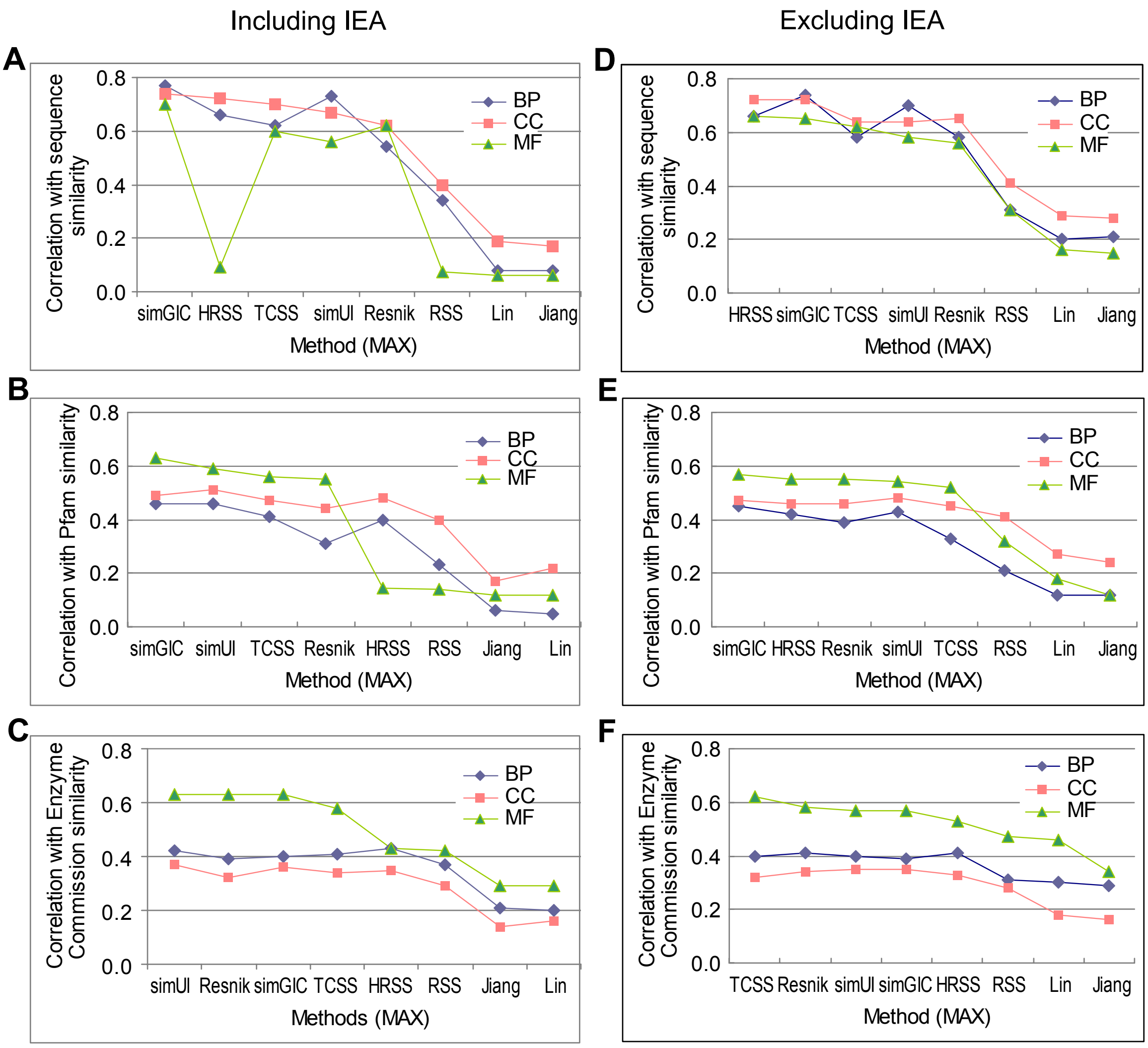

Figure S6. Correlation between semantic similarity (MAX) and the CESSM dataset (including and excluding IEA). CESSM holds the data of (**A** and **D**) sequence, (**B** and **E**) Pfam and (**C** and **F**) ECC similarities.
