# Supplementary material for: Improving the Measurement of Semantic Similarity between Gene Ontology Terms and Gene Products: Insights from an Edge- and IC-Based Hybrid Method
Source: PLoS One. 2013 May 31;8(5):e66745. doi: 10.1371/journal.pone.0066745 (PMC3669204; doi:10.1371/journal.pone.0066745)
Supplement: Figure S7 — Comparison of two pairwise strategies, MAX and BMA, on correlation with CESSM dataset (excluding IEA). The CESSM dataset shows the similarity of (A) sequence, (B) Pfam and (C) ECC for UniProt protein pairs. The difference between the two strategies was measured by the correlation coefficient of the BMA strategy minus that of MAX. (PDF) [file pone.0066745.s007.pdf]

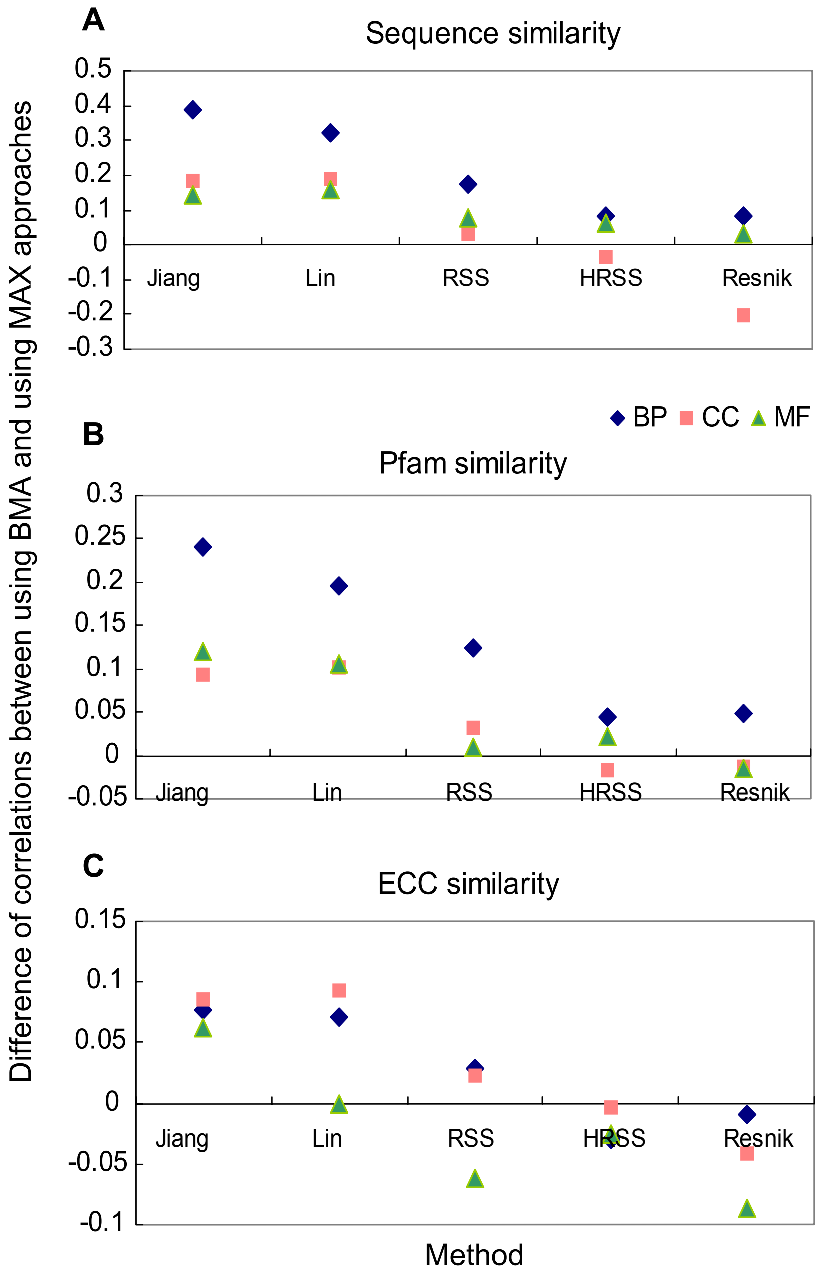

Figure S7. Comparison of two pairwise strategies, MAX and BMA, on correlation with CESSM dataset (excluding IEA). The CESSM dataset shows the similarity of (**A**) sequence, (**B**) Pfam and (**C**) ECC for UniProt protein pairs. The difference between the two strategies was measured by the correlation coefficient of the BMA strategy minus that of MAX.
