# Supplementary material for: Improving the Measurement of Semantic Similarity between Gene Ontology Terms and Gene Products: Insights from an Edge- and IC-Based Hybrid Method
Source: PLoS One. 2013 May 31;8(5):e66745. doi: 10.1371/journal.pone.0066745 (PMC3669204; doi:10.1371/journal.pone.0066745)
Supplement: Figure S8 — Correlation between semantic similarity and gene expression similarity. The (A and B) BMA and (C and D) MAX pairwise strategies were used. The evaluation based on the BP and MF ontologies (including IEA) was carried out for human and yeast, independently. (PDF) [file pone.0066745.s008.pdf]

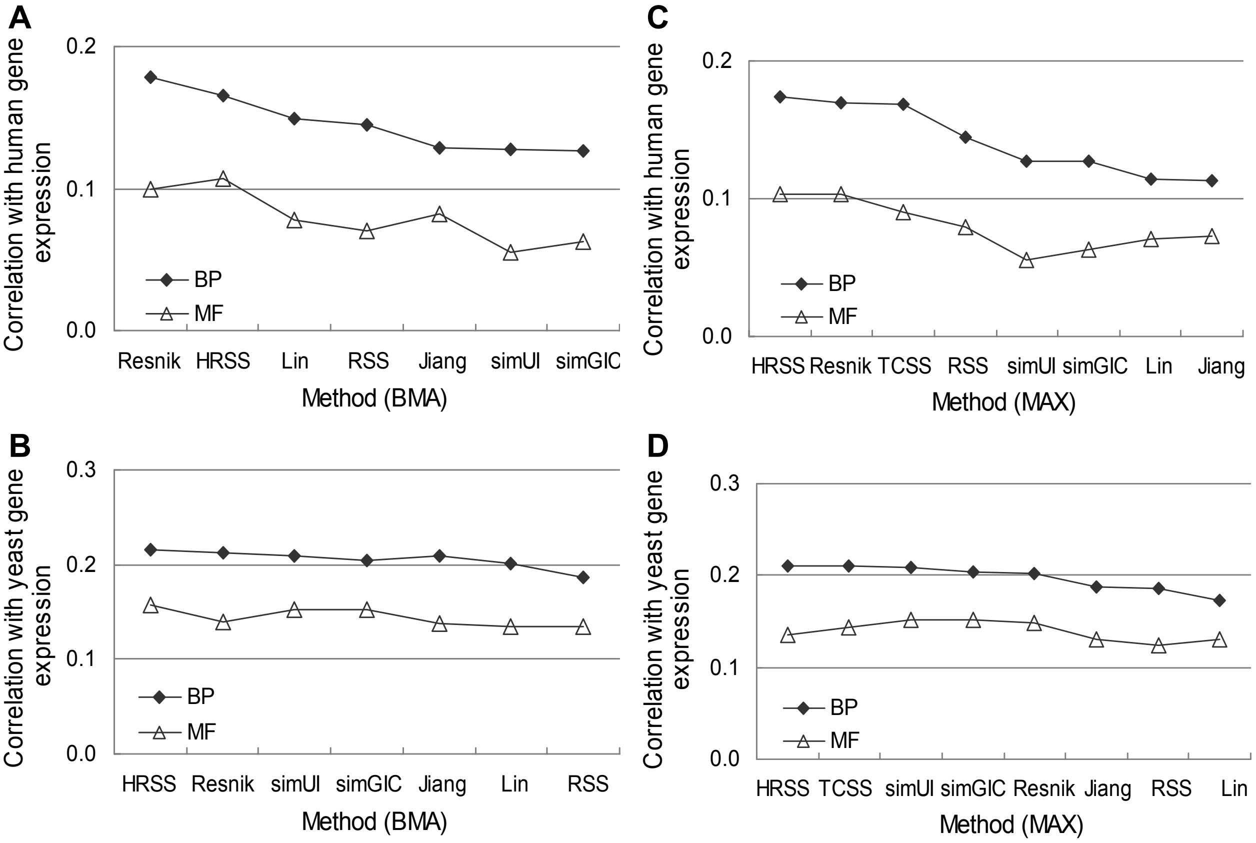

Figure S8. Correlation between semantic similarity and gene expression similarity. The (**A** and **B**) BMA and (**C** and **D**) MAX pairwise strategies were used. The evaluation based on the BP and MF ontologies (including IEA) was carried out for human and yeast, independently.
