# Supplementary material for: Improving the Measurement of Semantic Similarity between Gene Ontology Terms and Gene Products: Insights from an Edge- and IC-Based Hybrid Method
Source: PLoS One. 2013 May 31;8(5):e66745. doi: 10.1371/journal.pone.0066745 (PMC3669204; doi:10.1371/journal.pone.0066745)
Supplement: Table S1 — The sizes of gold standard positive and negative interaction datasets used for ROC analysis in human and yeast. (PDF) [file pone.0066745.s009.pdf]

Table S1. The sizes of gold standard positive and negative interaction datasets used for ROC analysis in human and yeast

	Human		Yeast	
GO	Including IEA	Including IEA	Including IEA	Excluding IEA
BP	2150	2016	4095	4061
CC	2179	1945	4129	4113
MF	2094	1842	3488	3203

Number of interactions in the positive and negative datasets for BB, CC and MF ontologies are listed. The negative dataset has the equal number of protein pairs as that in the positive dataset, and was generated by randomly choosing annotated proteins from a specific corpus of GO annotations that are absent from a set of all known genome-wide protein-protein interactions. The generation of negative datasets was done separately for three ontologies.
