# Supplementary material for: Improving the Measurement of Semantic Similarity between Gene Ontology Terms and Gene Products: Insights from an Edge- and IC-Based Hybrid Method
Source: PLoS One. 2013 May 31;8(5):e66745. doi: 10.1371/journal.pone.0066745 (PMC3669204; doi:10.1371/journal.pone.0066745)
Supplement: Table S2 — Area under the ROC curves (AUCs) for the yeast PPI dataset. (PDF) [file pone.0066745.s010.pdf]

Table S2. Area under the ROC curves (AUCs) for the yeast PPI dataset

		Including IEA			Excluding IEA		
		BP	CC	MF	BP	CC	MF
HRSS	MAX	**0.91**	**0.87**	**0.76**	**0.90**	**0.86**	0.72
	BMA	0.90	**0.87**	**0.76**	0.89	**0.86**	0.72
TCSS	MAX	0.90	0.85	**0.76**	**0.90**	0.85	0.73
Resnik	MAX	0.90	0.85	0.75	**0.90**	0.85	**0.74**
	BMA	0.89	0.85	0.75	0.89	0.84	0.72
RSS	MAX	0.89	0.85	0.75	0.89	0.84	0.72
	BMA	0.86	0.83	0.73	0.85	0.80	0.70
simGIC		0.87	0.80	**0.76**	0.87	0.79	0.72
simUI		0.84	0.77	**0.76**	0.84	0.76	0.72
Lin	MAX	0.82	0.65	0.72	0.89	0.71	0.73
	BMA	0.85	0.78	0.73	0.87	0.74	0.72
Jiang	MAX	0.81	0.65	0.68	0.85	0.69	0.70
	BMA	0.84	0.76	0.70	0.86	0.68	0.70

The tests were carried out separately for BP and CC ontologies. The MAX and BMA strategies were used for datasets including or excluding the IEA evidence code. For each group, the top scores are in bold.
