# Supplementary material for: Improving the Measurement of Semantic Similarity between Gene Ontology Terms and Gene Products: Insights from an Edge- and IC-Based Hybrid Method
Source: PLoS One. 2013 May 31;8(5):e66745. doi: 10.1371/journal.pone.0066745 (PMC3669204; doi:10.1371/journal.pone.0066745)
Supplement: Table S3 — Indexes used for evaluating the performance of HRSS (MAX) on scoring protein-protein interactions in yeast. (PDF) [file pone.0066745.s011.pdf]

Table S3. Indexes used for evaluating the performance of HRSS (MAX) on scoring protein-protein interactions in yeast

GO	HRSS	Predicted Positive	Predicted Negative	TP	FN	FP	TN	FPR (1-Specificity)	TPR (Sensitivity)	PPV	NPV	Youden index
BP	0.9	1002	7188	998	3097	4	4091	0.0010	0.2437	0.9960	0.5691	0.2427
	0.8	1119	7071	1115	2980	4	4091	0.0010	0.2723	0.9964	0.5786	0.2713
	0.7	1304	6886	1299	2796	5	4090	0.0012	0.3172	**0.9962**	0.5940	0.3160
	0.6	1559	6631	1548	2547	11	4084	0.0027	0.3780	0.9929	0.6159	0.3753
	0.5	1857	6333	1839	2256	18	4077	0.0044	0.4491	0.9903	0.6438	0.4447
	0.4	2315	5875	2274	1821	41	4054	0.0100	0.5553	0.9823	0.6900	0.5453
	0.3	2915	5275	2780	1315	135	3960	0.0330	0.6789	0.9537	0.7507	0.6459
	0.2	3517	4673	3218	877	299	3796	0.0730	0.7858	0.9150	0.8123	**0.7128**
	0.1	4885	3305	3629	466	1256	2839	0.3067	0.8862	0.7429	**0.8590**	0.5795
CC	0.9	1632	6626	1619	2510	13	4116	0.0031	0.3921	0.9920	0.6212	0.3890
	0.8	1665	6593	1652	2477	13	4116	0.0031	0.4001	0.9922	0.6243	0.3969
	0.7	1784	6474	1770	2359	14	4115	0.0034	0.4287	0.9922	0.6356	0.4253
	0.6	1870	6388	1854	2275	16	4113	0.0039	0.4490	0.9914	0.6439	0.4451
	0.5	1991	6267	1972	2157	19	4110	0.0046	0.4776	**0.9905**	0.6558	0.4730
	0.4	2212	6046	2171	1958	41	4088	0.0099	0.5258	0.9815	0.6761	0.5159
	0.3	2750	5508	2560	1569	190	3939	0.0460	0.6200	0.9309	0.7151	0.5740
	0.2	3452	4806	2912	1217	540	3589	0.1308	0.7053	0.8436	0.7468	**0.5745**
	0.1	5354	2904	3671	458	1683	2446	0.4076	0.8891	0.6857	**0.8423**	0.4815

4095 and 4129 protein pairs are in the positive protein-protein interaction datasets for BP and CC ontologies, respectively. The negative dataset contains the same number of protein pairs as that in positive dataset. The HRSS thresholds chosen for defining positive and negative interactions were highlighted in gray. PPV was used as one index for selecting positive interactions while NPV was mainly for selecting negative ones.
