# Supplementary material for: Improving the Measurement of Semantic Similarity between Gene Ontology Terms and Gene Products: Insights from an Edge- and IC-Based Hybrid Method
Source: PLoS One. 2013 May 31;8(5):e66745. doi: 10.1371/journal.pone.0066745 (PMC3669204; doi:10.1371/journal.pone.0066745)
Supplement: Table S4 — Indexes used for evaluating the performance of HRSS (MAX) on scoring protein-protein interactions in human. (PDF) [file pone.0066745.s012.pdf]

Table S4. Indexes used for evaluating the performance of HRSS (MAX) on scoring protein-protein interactions in human

GO	HRSS	Predicted Positive	Predicted Negative	TP	FN	FP	TN	FPR (1-Specificity)	TPR (Sensitivity)	PPV	NPV	Youden index
BP	0.9	409	3891	409	1741	0	2150	0.0000	0.1902	1.0000	0.5526	0.1902
	0.8	440	3860	439	1711	1	2149	0.0005	0.2042	0.9977	0.5567	0.2037
	0.7	534	3766	533	1617	1	2149	0.0005	0.2479	0.9981	0.5706	0.2474
	0.6	729	3571	726	1424	3	2147	0.0014	0.3377	**0.9959**	0.6012	0.3363
	0.5	982	3318	970	1180	12	2138	0.0056	0.4512	0.9878	0.6444	0.4456
	0.4	1112	3188	1090	1060	22	2128	0.0102	0.5070	0.9802	0.6675	0.4967
	0.3	1499	2801	1421	729	78	2072	0.0363	0.6609	0.9480	0.7397	0.6247
	0.2	1789	2511	1618	532	171	1979	0.0795	0.7526	0.9044	0.7881	**0.6730**
	0.1	2685	1615	1950	200	735	1415	0.3419	0.9070	0.7263	**0.8762**	0.5651
CC	0.9	355	4003	350	1829	5	2174	0.0023	0.1606	0.9859	0.5431	0.1583
	0.8	367	3991	362	1817	5	2174	0.0023	0.1661	0.9864	0.5447	0.1638
	0.7	406	3952	400	1779	6	2173	0.0028	0.1836	0.9852	0.5498	0.1808
	0.6	433	3925	427	1752	6	2173	0.0028	0.1960	0.9861	0.5536	0.1932
	0.5	483	3875	476	1703	7	2172	0.0032	0.2184	**0.9855**	0.5605	0.2152
	0.4	631	3727	613	1566	18	2161	0.0083	0.2813	0.9715	0.5798	0.2731
	0.3	831	3527	785	1394	46	2133	0.0211	0.3603	0.9446	0.6048	0.3391
	0.2	1747	2611	1478	701	269	1910	0.1235	0.6783	0.8460	0.7315	**0.5548**
	0.1	2671	1687	1893	286	778	1401	0.3570	0.8687	0.7087	**0.8305**	0.5117

2150 and 2179 protein pairs are in the positive protein-protein interaction datasets BP and CC ontologies, respectively. The negative dataset contains the same number of protein pairs as that in positive dataset. The HRSS thresholds chosen for defining positive and negative interactions were highlighted in gray. PPV was used as one index for selecting positive interactions while NPV was mainly for selecting negative ones.
