# Supplementary material for: Improving the Measurement of Semantic Similarity between Gene Ontology Terms and Gene Products: Insights from an Edge- and IC-Based Hybrid Method
Source: PLoS One. 2013 May 31;8(5):e66745. doi: 10.1371/journal.pone.0066745 (PMC3669204; doi:10.1371/journal.pone.0066745)
Supplement: Table S5 — Summary of the functional similarities of observed human-mouse orthologs calculated by various semantic similarity methods (including IEA). (PDF) [file pone.0066745.s013.pdf]

Table S5. Summary of the functional similarities of observed human-mouse orthologs calculated by various semantic similarity methods (including IEA)

Strategy	GO	Num. of orthologs	Functional similarity	HRSS	simGIC	simUI	Resnik	TCSS	Jiang	Lin	RSS
BMA	BP	5726	Mean	0.45	0.76	0.79	2.95		0.89	0.92	0.79
			Stdev.[a]	0.1721	0.2804	0.2516	0.8803		0.1736	0.1332	0.1107
			SE[b]	0.0023	0.0037	0.0033	0.0116		0.0023	0.0018	0.0015
	CC	5539	Mean	0.30	0.81	0.86	1.85		0.97	0.97	0.79
			Stdev.	0.1535	0.2664	0.1982	0.7624		0.0870	0.0769	0.0954
			SE	0.0021	0.0036	0.0027	0.0102		0.0012	0.0010	0.0013
	MF	4966	Mean	0.46	0.85	0.87	2.32		0.94	0.94	0.79
			Stdev.	0.1908	0.2415	0.2009	0.8771		0.1351	0.1252	0.1256
			SE	0.0027	0.0034	0.0029	0.0124		0.0019	0.0018	0.0018
MAX	BP	5726	Mean	0.81	0.76	0.79	4.63	0.68	0.99	1.00	0.94
			Stdev.	0.2398	0.2804	0.2516	1.1050	0.1730	0.0795	0.0544	0.0803
			SE	0.0032	0.0037	0.0033	0.0146	0.0023	0.0011	0.0007	0.0011
	CC	5539	Mean	0.62	0.81	0.86	3.29	0.68	1.00	1.00	0.91
			Stdev.	0.3282	0.2664	0.1982	1.3522	0.3297	0.0430	0.0409	0.0866
			SE	0.0044	0.0036	0.0027	0.0182	0.0044	0.0006	0.0005	0.0012
	MF	4966	Mean	0.85	0.85	0.87	3.96	0.58	0.99	0.99	0.94
			Stdev.	0.2786	0.2415	0.2009	1.4119	0.2092	0.0900	0.0840	0.1274
			SE	0.0040	0.0034	0.0029	0.0200	0.0030	0.0013	0.0012	0.0018

[a] Standard deviation (stdev.) of the functional similarities of observed orthologs.

[b] Standard error (SE) calculated as stdev./sqrt(N) where N is the number of orthologs.
