# Supplementary material for: Improving the Measurement of Semantic Similarity between Gene Ontology Terms and Gene Products: Insights from an Edge- and IC-Based Hybrid Method
Source: PLoS One. 2013 May 31;8(5):e66745. doi: 10.1371/journal.pone.0066745 (PMC3669204; doi:10.1371/journal.pone.0066745)
Supplement: Table S6 — Z-score analysis of various semantic similarity methods (BMA) for estimating the functional similarity among human-mouse orthologs. (PDF) [file pone.0066745.s014.pdf]

Table S6. Z-score analysis of various semantic similarity methods (BMA) for estimating the functional similarity among human-mouse orthologs

IEA	GO	ASV[a]	HRSS	simGIC	simUI	Resnik	Jiang	Lin	RSS
Including	BP	Observed[b]	0.45	0.76	0.79	2.95	0.89	0.92	0.79
		Mean. randomized[c]	0.04	0.05	0.10	0.86	0.19	0.36	0.41
		Stdev. Randomized[d]	0.0003	0.0007	0.0010	0.0055	0.0020	0.0023	0.0014
		Z-score[e]	**1341.01**	1100.88	727.86	381.64	349.16	246.14	273.49
	CC	Observed	0.30	0.81	0.86	1.85	0.97	0.97	0.79
		Mean. Randomized	0.05	0.13	0.28	0.63	0.55	0.58	0.54
		Stdev. Randomized	0.0005	0.0015	0.0017	0.0038	0.0026	0.0027	0.0014
		Z-score	**528.09**	465.02	348.29	322.54	159.10	147.01	169.36
	MF	Observed	0.46	0.85	0.87	2.32	0.94	0.94	0.79
		Mean. Randomized	0.06	0.05	0.11	0.47	0.28	0.33	0.34
		Stdev. Randomized	0.0006	0.0013	0.0014	0.0042	0.0029	0.0029	0.0020
		Z-score	**643.27**	615.01	566.87	436.91	227.51	210.96	230.78
Excluding	BP	Observed	0.31	0.48	0.52	1.77	0.58	0.71	0.65
		Mean. Randomized	0.04	0.04	0.08	0.55	0.06	0.27	0.36
		Stdev. Randomized	0.0003	0.0008	0.0010	0.0035	0.0015	0.0018	0.0014
		Z-score	**861.73**	547.18	451.77	347.74	349.43	246.00	206.08
	CC	Observed	0.25	0.58	0.67	1.29	0.75	0.83	0.73
		Mean. Randomized	0.04	0.13	0.27	0.47	0.31	0.45	0.53
		Stdev. Randomized	0.0005	0.0017	0.0019	0.0030	0.0030	0.0026	0.0014
		Z-score	**423.44**	269.83	210.94	272.64	149.10	145.80	142.33
	MF	Observed	0.40	0.60	0.64	1.72	0.79	0.82	0.68
		Mean. Randomized	0.05	0.05	0.11	0.35	0.31	0.36	0.29
		Stdev. Randomized	0.0006	0.0015	0.0017	0.0039	0.0026	0.0027	0.0023
		Z-score	**592.07**	357.11	307.03	355.88	188.14	167.54	169.02

[a] To measure the functional similarity of a list of orthologous protein pairs in general, an average value was calculated from the similarity values (ASV) of the orthologs.

[b] ASV value calculated from the observed set of human-mouse orthologs, i.e., $ASV_{observed}$.

[c] Mean ASVs calculated from 1000 randomized sets of orthologs, i.e., $mean(ASV_{random})$.

[d] Standard deviation (stdev.) of ASVs from 1000 randomized sets of orthologs, i.e.,

$stdev(ASV_{random})$.

[e] Z-score was calculated as $(ASV_{observed}-mean(ASV_{random}))/stdev(ASV_{random})$. The maximum Z-score for each ontology is in bold.
