# Supplementary material for: Improving the Measurement of Semantic Similarity between Gene Ontology Terms and Gene Products: Insights from an Edge- and IC-Based Hybrid Method
Source: PLoS One. 2013 May 31;8(5):e66745. doi: 10.1371/journal.pone.0066745 (PMC3669204; doi:10.1371/journal.pone.0066745)
Supplement: Table S7 — Z-score analysis of various semantic similarity methods (MAX) for estimating the functional similarity among human-mouse orthologs. (PDF) [file pone.0066745.s015.pdf]

Table S7. Z-score analysis of various semantic similarity methods (MAX) for estimating the functional similarity among human-mouse orthologs

IEA	GO	ASV[a]	HRSS	simGIC	simUI	Resnik	TCSS	Jiang	Lin	RSS
Including	BP	Observed[b]	0.81	0.76	0.79	4.63	0.68	0.99	1.00	0.94
		Mean. randomized[c]	0.12	0.05	0.10	1.83	0.27	0.57	0.73	0.65
		Stdev. Randomized[d]	0.0013	0.0007	0.0010	0.0097	0.0014	0.0043	0.0031	0.0016
		Z-score[e]	525.72	**1100.88**	727.86	287.21	282.39	98.86	86.10	173.64
	CC	Observed	0.62	0.81	0.86	3.29	0.68	1.00	1.00	0.91
		Mean. Randomized	0.12	0.13	0.28	1.14	0.16	0.85	0.85	0.73
		Stdev. Randomized	0.0013	0.0015	0.0017	0.0081	0.0015	0.0028	0.0028	0.0015
		Z-score	391.83	**465.02**	348.29	264.78	348.22	53.98	54.82	116.78
	MF	Observed	0.85	0.85	0.87	3.96	0.58	0.99	0.99	0.94
		Mean. Randomized	0.17	0.05	0.11	1.00	0.15	0.62	0.66	0.61
		Stdev. Randomized	0.0023	0.0013	0.0014	0.0080	0.0012	0.0045	0.0039	0.0024
		Z-score	297.92	**615.01**	566.87	369.60	360.44	82.06	86.08	136.83
Excluding	BP	Observed	0.57	0.48	0.52	2.71	0.53	0.86	0.91	0.84
		Mean. Randomized	0.08	0.04	0.08	0.96	0.19	0.23	0.50	0.54
		Stdev. Randomized	0.0009	0.0008	0.0010	0.0059	0.0012	0.0041	0.0029	0.0018
		Z-score	532.61	**547.18**	451.77	295.99	292.45	152.50	139.96	163.13
	CC	Observed	0.42	0.58	0.67	1.85	0.48	0.91	0.94	0.83
		Mean. Randomized	0.08	0.13	0.27	0.64	0.12	0.51	0.63	0.63
		Stdev. Randomized	0.0009	0.0017	0.0019	0.0046	0.0011	0.0043	0.0033	0.0017
		Z-score	**376.18**	269.83	210.94	266.61	337.60	93.33	94.95	120.32
	MF	Observed	0.60	0.60	0.64	2.40	0.48	0.93	0.94	0.83
		Mean. Randomized	0.08	0.05	0.11	0.52	0.11	0.49	0.54	0.44
		Stdev. Randomized	0.0013	0.0015	0.0017	0.0059	0.0012	0.0035	0.0034	0.0029
		Z-score	**411.69**	357.11	307.03	318.32	309.14	126.14	118.17	131.37

The detail description is the same as that in **Table S6**.
