# Supplementary material for: Improving the Measurement of Semantic Similarity between Gene Ontology Terms and Gene Products: Insights from an Edge- and IC-Based Hybrid Method
Source: PLoS One. 2013 May 31;8(5):e66745. doi: 10.1371/journal.pone.0066745 (PMC3669204; doi:10.1371/journal.pone.0066745)
Supplement: Text S1 — Describes additional details of the semantic similarity methods used in this study, as well as additional information regarding the correlation between semantic similarities and gene co-expression. (PDF) [file pone.0066745.s016.pdf]

Improving the Measurement of Semantic Similarity between Gene Ontology Terms and Gene Products: Insights from an Edge- and IC-based Hybrid Method

Xiaomei Wu, Erli Pang, Kui Lin, Zhen-Ming Pei

Supplementary Text S1

Contents

Section S1. Definition of Relative Specificity Similarity (RSS) method 2
Section S2. Definition of the semantic similarity methods used in the study 2
Section S3. Poor correlations of semantic similarities with gene co-expression 5
References 6

Section S1. Definition of Relative Specificity Similarity (RSS) method

In 2006, we designed a metric for evaluating the relative specificity semantic similarity between two GO terms, and named it as RSS. We scored the functional similarity of two proteins by considering the maximum RSS values of all term pairs [1]. For a given GO, let $term_i$ and $term_j$ be two terms, and $Paths(term_i)$ and $Paths(term_j)$ be the paths in the graphs induced from $term_i$ and $term_j$, respectively, to the root term of the GO. We defined $dist(term_i, term_j)$ as the number of edges along the shortest path between $term_i$ and $term_j$, such that the value equals zero if the two terms are the same. The RSS of the two GO terms, $term_i$ and $term_j$ consists of three different components (Figure 1A), denoted α, β and γ. Component α is defined in Formula 1 and is equivalent to the definition of S in Wu's work [2]. It measures how specific the most recent common ancestor (MRCA) of the two terms is according to the structure of the GO.

$$a = \max_{\substack{path_m \in Paths(term_i), \\ path_n \in Paths(term_j)}} \left\{ \begin{array}{c} \text{the number of common terms} \\ \text{between } path_m \text{ and } path_n \end{array} \right\} - 1 \quad (1)$$

Obviously, the larger component α is, the more specific the MRCA.

Component β measures how general $term_i$ and $term_j$ are in the GO and is defined in Formula 2. The generality of a term is defined as the minimum distance between the term and the leaf terms descending from it. Leaf terms in a GO are those terms without any descendant. Obviously, the larger the distance between a term and its leaves, the more general is the term.

$$\beta = \max\{\min_{u \in U}\{dist(term_i, u)\}, \min_{v \in V}\{dist(term_j, v)\}\} \quad (2)$$

where U and V indicate all leaf nodes descending from $term_i$ and $term_j$, respectively.

Component γ measures the local distance between two terms and the MRCA and is defined as

$$\gamma = dist(MRCA, term_i) + dist(MRCA, term_j)\,. \quad (3)$$

If γ is smaller, it implies $term_i$ and $term_j$ share more similarity locally relative to the MRCA.

Then, the RSS between two terms of a given GO, $term_i$ and $term_j$ can be quantified by combining α, β and γ together in Formula 4,

$$RSS(term_i, term_j) = \frac{maxDepth^{GO}}{maxDepth^{GO} + \gamma} \cdot \frac{\alpha}{\alpha + \beta} \quad (4)$$

where $maxDepth^{GO}$ is the maximum distance from the root term of the GO to the leaf terms. From the definition, the values of RSS are between 0 and 1. Clearly, RSS = 0 ($\alpha = 0$) indicates that the MRCA of $term_i$ and $term_j$ is the root of the GO, which means that the two terms share no commonality in describing protein properties; on the other hand, RSS = 1 ($\gamma = 0$ and $\beta = 0$) indicates that $term_i$ and $term_j$ are the same leaf term, which means that the two terms are most specific in describing protein attributes. RSS calculates semantic similarity not only takes the specificity of a common ancestor (α) into account, but also considers the position in the global GO DAG where any two terms are (β), as well as the local similarity between the term pair and their MRCA.

Section S2. Definition of the semantic similarity methods used in the study

Six semantic similarity methods were compared with RSS and HRSS in the evaluation analyses. RSS, HRSS, Resnik [3], Jiang [4], Lin [5] and TCSS [6] are node-based methods that use pairwise approaches, while simUI [7] and simGIC [8] are groupwise measures. RSS and HRSS methods were implemented using C programming language. Resnik, Jiang, Lin, simUI and simGIC were

also implemented in our study as described in their respective publications. TCSS was computed using the program provided by the publication [6]. Both maximum (MAX) and best-match average (BMA) strategies were used to compare the functional similarity of pairwise term pairs annotated on two proteins. The software of TCSS only provides the results of MAX strategy. simUI and simGIC consider the sets of GO terms for two proteins and uses the Jaccard index to calculate the similarity between them, thus MAX and BMA strategies are not relevant for them.

Most of node-based methods are based on information content (IC) that estimates the property of a term *c*, and measures how specific and informative the term is. IC is commonly defined as the negative log likelihood of the term,

$$IC(c) = -\log p(c) \tag{5}$$

where *p(c)* is the probability of occurrence of the term *c* in a specific corpus (such as the GO annotations of yeast genome or UniProt Knowledgebase), and is normally measured by the frequency of annotations on *c* and all the descendents in the sub-DAG rooted from *c*. The more often the term is used for annotation, the lower its semantic value.

Resnik [3] defined a semantic similarity between two terms c_1 and c_2 as simply the IC of their most informative common ancestor (MICA),

$$sim_{Resnik}(c_1, c_2) = IC(MICA)\,. \tag{6}$$

Jiang and Conrath [4] proposed a hybrid semantic similarity measure that inherits from the edge-based method and weights each edge by several factors, such as difference in IC, local density, node depth, and link type. The edge weight (*wt*) for a child node *c* and its parent node *p* is in Formula 7,

$$wt(cp) = (\beta + (1-\beta)\frac{\overline{E}}{E(p)})(\frac{d(p)+1}{d(p)})^{\alpha}[IC(c) - IC(p)]T(c,p) \tag{7}$$

where *d(p)* denotes the depth of the node *p* (usually calculated as the longest path length from the root of the DAG to *p*), *E(p)* the number of edges in the child links (i.e. local density), $\overline{E}$ the average density in the whole DAG, and *T(c,p)* the link relation/type factor. Two weighting factors, α ($\alpha \geqslant 0$) and β ($0 \leqslant \beta \geqslant 1$) control the degree of how much the node depth and local density contribute to the edge weighting computation. Note that these contributions become less significant when α approaches 0 and β approaches 1.

Then the overall distance between a node *c* and one of its ancestor (*ance*) is defined as the summation of edge weights along the shortest path linking them (*path(c,ance)*),

$$d(c, ance) = \sum_{c_i \in path(c,ance)} wt(c_i, parent(c_i))\,. \tag{8}$$

Now the semantic similarity between any two nodes (c_1 and c_2) relative to their MICA is defined as,

$$d(c_1, c_2) = dist(c_1, MICA) + dist(c_2, MICA)\,. \tag{9}$$

In the special case, where only IC is considered while factors related to node depth, local density and link type are ignored, i.e., $\alpha = 0$, $\beta = 1$ and *T(c,a)* = 1, the distance between the two nodes can be simplified as,

$$d_{Jiang}(c_1, c_2) = IC(c_1) + IC(c_2) - 2IC(MICA)\,. \tag{10}$$

The simplified semantic distance could be converted to a similarity using the formula in [9]

$$sim_{Jiang}(c_1,c_2)=1-\min(1,d_{Jiang}(c_1,c_2)). \qquad (11)$$

Jiang and Conrath showed that their measure is not very sensitive to changes in the values of α and β. Hence the node depth and local density are not the major determinants of the overall edge weight [4].

Lin [5] considered the distance of the terms from their common ancestor in a different way,

$$sim_{Lin}(c_1,c_2)=\frac{2IC(MICA)}{IC(c_1)+IC(c_2)}. \qquad (12)$$

Resnik, Jiang and Lin are the most commonly used IC-based semantic similarity measures. But they do not consider the unequal depth of biological knowledge representation in different braches of the GO graph. To overcome this, Jain and Bader [6] designed an improved IC-based algorithm, Topological Clustering Semantic Similarity (TCSS) by clustering similar GO terms into sub-graphs. A meta-graph was firstly created by partitioning the GO DAG into non-overlapping sub-graphs. Then, a semantic similarity between two GO terms s_i and t_j was calculated based on the annotation information content (ICA) of their MICA. If s_i and t_j belong to the same sub-graph, then their MICA will be in that sub-graph. The TCSS value of s_i and t_j is defined as

$$TCSS(s_i,t_j)=ICS_{\max}(MICA). \qquad (13)$$

ICS (sub-graph information content) is a normalized value like

$$ICS(t_i^s)=\frac{ICA(t_i^s)}{\max\limits_{t_i^s\in G_i^s} ICA(t_i^s)} \qquad (14)$$

where the term t_i^s belongs to the ith sub-graph G_i^s. If s_i and t_j belong to the different sub-graphs, then their MICA will be belong to the meta-graph,

$$TCSS(s_i,t_j)=ICM_{\max}(MICA). \qquad (15)$$

ICM (meta-graph information content) of a term t_i^m in meta-graph G^m is calculated within the meta-graph,

$$ICM(t_i^m)=\frac{ICA(t_i^m)}{\max\limits_{t_i^m\in G^m} ICA(t_i^m)}. \qquad (16)$$

Let *P* and *Q* be two gene products of interest, and *TP* and *TQ* the sets of all the GO terms assigned to protein *P* and *Q*, respectively. Two pairwise approaches, namely MAX and BMA were implemented to quantify the relationship strength between *P* and *Q*. The MAX approach calculates the maximum semantic similarity score among all pairs of GO terms between *TP* and *TQ*,

$$sim_{MAX}^{GO}(P,\ Q)=\max_{\substack{tp_i\in TP\\ tq_j\in TQ}}\{sim(tp_i,tq_j)\}. \qquad (17)$$

The BMA approach computes the average of all maximum similarities for each term in *TP* and *TQ*,

$$sim_{BMA}^{GO}(P,\ Q) = \frac{\sum_{tp_i \in TP} sim(tp_i, TQ) + \sum_{tq_j \in TQ} sim(tq_j, TP)}{|TP| + |TQ|} \quad (18)$$

where $sim(u_i, V) = \max_{v_j \in V}\{sim(u_i, v_j)\}$.

Different from the aforementioned pairwise approaches, simUI [7] and simGIC [8] calculate the semantic similarity between two gene products based on measuring the two sets of annotated terms. Given two gene products *P* and *Q*, *Terms*(*P*) and *Terms*(*Q*) are extended annotations sets of *P* and *Q*, respectively. *Terms*(*P*) includes both direct GO annotations of protein *P* and all their ancestral terms up to the root term of the GO. Using the Jaccard index, simUI defines the similarity between the two proteins as the number of terms in the intersection of *Terms*(*P*) and *Terms*(*Q*) divided by the number of terms in the union,

$$simUI(P,Q) = \frac{|Terms(P) \cap Terms(Q)|}{|Terms(P) \cup Terms(Q)|} \quad (19)$$

simGIC proposed a weighted Jaccard index where each GO term is weighted by its IC. The simGIC value between *P* and *Q* is measured as the sum of the IC of each term in the intersection of *Terms*(*P*) and *Terms*(*Q*) divided by that in their union,

$$simGIC(P,Q) = \frac{\sum_{c \in Terms(P) \cap Terms(Q)} IC(c)}{\sum_{c \in Terms(P) \cup Terms(Q)} IC(c)}. \quad (20)$$

Section S3. Poor correlations of semantic similarities with gene co-expression

We tested the performance of GO-based semantic similarity measures on the correlation with gene co-expression. First, two expression compendiums (tissue-specific pattern of mRNA expression in human and yeast cell cycle) were prepared as followers. (1) Human microarray data presented in Su *et al.* [10] was normalized and parsed by Nehrt et al. [11]. We were able to obtain expression data for 14,987 human genes in 25 tissues. Like Jain et al. [6], tests datasets for the three ontologies (including IEA annotations) were built independently by randomly selecting 3400 yeast protein pairs in the combined gene expression dataset, including an equal number of known protein-protein interactions (from human positive PPI dataset) and random protein pairs. (2) Four yeast cell cycle datasets presented in Spellman *et al.* [12] were retrieved from the Gene Expression Omnibus in NCBI with accessions GSE22 (Alpha-factor block-release), GSE23 (cdc15 block-release), GSE24 (Elutriation time course) and GSE25 (Cyclin overexpression). Expression data was normalized within a single sample in each experiment dataset using Z-score method based on the original log2 fold change values, forcing expression values within a sample to have a mean of 0 and a standard deviation of 1. Then, an expression compendium with 6035 yeast genes in 60 samples was obtained by combining the four normalized experiment datasets. Test datasets for the three ontologies (including IEA) were built independently by randomly choosing 6000 yeast protein pairs in the expression dataset, including an equal number of known protein-protein interactions and random protein pairs.

Next, Pearson's correlation coefficients were computed to quantify the relationship between semantic similarity and gene co-expression on the BP and MF ontologies. As shown in Figure S8, the correlation coefficients are fairly low, only with the maximum value of 0.2, indicating poor

linear correlations between semantic similarity and expression similarity. We also computed the Spearman's rank correlation rho and obtained similar correlations (data not shown).

References

1. Wu X, Zhu L, Guo J, Zhang DY, Lin K (2006) Prediction of yeast protein-protein interaction network: insights from the Gene Ontology and annotations. Nucleic Acids Res 34: 2137-2150.
2. Wu H, Su Z, Mao F, Olman V, Xu Y (2005) Prediction of functional modules based on comparative genome analysis and Gene Ontology application. Nucleic Acids Res 33: 2822-2837.
3. Resnik P (1995) Using Information Content to Evaluate Semantic Similarity in a Taxonomy. IJCAI'95: Proceedings of the 14th International Joint Conference on Artificial Intelligence San Francisco, CA, USA.
4. Jiang JJ, Conrath DW (1997) Semantic Similarity Based on Corpus Statistics and Lexical Taxonomy. Proceedings of International Conference Research on Computational Linguistics (ROCLING X): 9008.
5. Lin D (1998) An Information-Theoretic Definition of Similarity. Proceedings of the Fifteenth International Conference on Machine Learning: Morgan Kaufmann Publishers Inc. pp. 296-304.
6. Jain S, Bader GD (2010) An improved method for scoring protein-protein interactions using semantic similarity within the Gene Ontology. BMC Bioinformatics 11: 562.
7. Gentleman R (2005) Visualizing and Distances Using GO. URL http://wwwbioconductororg/docs/vignetteshtml.
8. Pesquita C, Faria D, Bastos H, Falcao AO, Couto FM (2007) Evaluating GO-based Semantic Similarity Measures. In: ISMB/ECCB 2007 SIG Meeting Program Materials International Society for Computational Biology.
9. Yu G, Li F, Qin Y, Bo X, Wu Y, et al. (2010) GOSemSim: an R package for measuring semantic similarity among GO terms and gene products. Bioinformatics 26: 976-978.
10. Su AI, Wiltshire T, Batalov S, Lapp H, Ching KA, et al. (2004) A gene atlas of the mouse and human protein-encoding transcriptomes. Proc Natl Acad Sci U S A 101: 6062-6067.
11. Nehrt NL, Clark WT, Radivojac P, Hahn MW (2011) Testing the ortholog conjecture with comparative functional genomic data from mammals. PLoS Comput Biol 7: e1002073.
12. Spellman PT, Sherlock G, Zhang MQ, Iyer VR, Anders K, et al. (1998) Comprehensive identification of cell cycle-regulated genes of the yeast Saccharomyces cerevisiae by microarray hybridization. Mol Biol Cell 9: 3273-3297.
